# A STAT3/integrin axis accelerates pancreatic cancer initiation and progression

**DOI:** 10.1016/j.celrep.2025.116010

**Published:** 2025-07-22

**Authors:** Alejandro D. Campos, Ryan M. Shepard, Zachary Ortega, Ingrid Heumann, Anna E. Wilke, Arin Nam, Carson Cable, Kourosh Kouhmareh, Richard Klemke, Nicole M. Mattson, Trey Ideker, Camila De Arruda Saldanha, Sven Heinz, Valerie Weaver, Tami Von Schalscha, Hiromi I. Wettersten, Sara M. Weis, David A. Cheresh

**Affiliations:** 1 Department of Pathology, Moores Cancer Center at the University of California San Diego, La Jolla, San Diego, CA, USA; 2 Department of Pathology, Sanford Consortium for Regenerative Medicine at the University of California San Diego, La Jolla, San Diego, CA, USA; 3 Division of Genomics and Precision Medicine, Department of Medicine, University of California San Diego, La Jolla, San Diego, CA, USA; 4 Departments of Bioengineering and Computer Science and Engineering, University of California San Diego, La Jolla, San Diego, CA, USA; 5 School of Medicine, University of California San Diego, La Jolla, San Diego, CA, USA; 6 Department of Surgery and Center for Bioengineering and Tissue Regeneration, University of California San Francisco, San Francisco, CA, USA; 7 These authors contributed equally; 8 Lead contact

## Abstract

The signal transducer and activator of transcription 3 (STAT3) pathway drives pancreatic ductal adenocarcinoma (PDAC) progression by coordinating cellular responses to stress and inflammation. We perform ChIP-seq on hypoxia- or oncostatin-*M*-treated PDAC cells to identify sites at which phospho-STAT3 binds to regulate the expression of genes linked to poor survival. A top hit among these is *ITGB3*, which we show promotes PDAC initiation and progression. Single-cell transcriptomics reveal that *ITGB3* expression is enriched in PDAC cells experiencing oxidative stress due to chemotherapy. Moreover, high *ITGB3* expression positively correlates with STAT3 signaling, hypoxia, and the basal subtype. Mechanistically, chromatin accessibility at *ITGB3* enhancers controls STAT3’s ability to induce *ITGB3* expression, illuminating a plastic regulatory mechanism modulating STAT3 activity. Leveraging this insight, we identify additional STAT3 target genes regulated similarly to *ITGB3* to establish an 18-gene signature involved in adaptive responses and able to stratify survival outcomes. Collectively, these findings highlight a novel opportunity to stratify PDAC subpopulations for STAT3-targeted therapies.

## In brief

Campos and Shepard et al. report that hypoxia and inflammatory cytokines activate the STAT3/integrin β3 axis, promoting stress tolerance and accelerating pancreatic cancer initiation and progression. Chromatin accessibility modulates STAT3 activity, revealing a gene expression pattern able to stratify patient survival outcomes and identify tumors likely to respond to STAT3-targeted therapies.

## Graphical Abstract

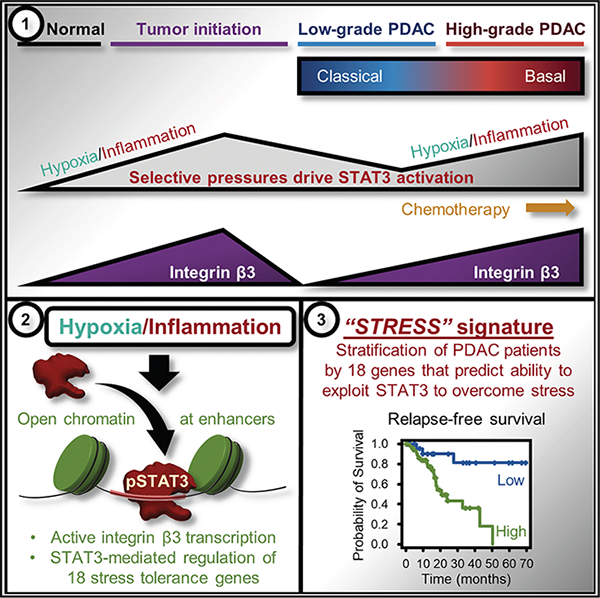

## INTRODUCTION

Inflammation and cellular stress, such as hypoxia and oxidative stress, are intrinsic to tumor microenvironments and are particularly prevalent in pancreatic ductal adenocarcinoma (PDAC).^1,2^ Importantly, both stress and inflammation have been reported to promote tumor initiation, progression, and drug resistance.^3,4^ The Janus kinase (JAK) and signal transducer and activator of transcription 3 (STAT3) pathway facilitates cellular responses to extracellular signals such as cytokines, chemokines, and various forms of cellular stress. Therefore, JAK-STAT3 signaling is critical for adaptive responses to stimuli readily present in the tumor microenvironment. STAT3-dependent transcription has been associated with the progression of multiple cancers, including PDAC.^5,6^ Notably, elevated levels of JAK-STAT3 signaling are associated with pancreatic cancer, and STAT3 activity has been reported to drive tumor initiation, progression, and therapeutic resistance in PDAC.^7,8^

We aimed to identify STAT3 target genes regulated in response to hypoxic stress and inflammatory cytokines commonly associated with the PDAC microenvironment. Although STAT3 can regulate hundreds of genes, we hypothesized that these stress and inflammatory response genes will mediate STAT3’s core oncogenic activities and provide therapeutic opportunities. To identify critical STAT3 target genes, we investigated genome-wide changes in STAT3-DNA binding following exposure of PDAC cells to inflammatory cytokines or hypoxia and identified *ITGB3* as a critical STAT3 response gene. Interestingly, integrin β3, the *ITGB3* gene product, and its functional heterodimer integrin αvβ3 have also been reported to drive tumor initiation, drug resistance, and metastasis, similarly to STAT3,^9–11^ suggesting an important JAK/STAT3/integrin β3 signaling axis in PDAC.

We report here that this JAK1/STAT3/integrin β3 axis accelerates PDAC initiation and progression and demonstrate STAT3 as necessary for stress- and inflammation-induced β3 expression. Additionally, we profile single-cell RNA sequencing (scRNA-seq) from patients with PDAC and observe that *ITGB3* expression is enriched in patients treated with chemotherapy relative to patients naive to treatment. Moreover, high *ITGB3* expression positively correlates with activated STAT3, hypoxia and cytokine signaling, and Moffitt’s Basal molecular subtype gene signature. Importantly, we demonstrate that the upregulation of *ITGB3* requires accessible chromatin at *ITGB3* enhancers to allow for STAT3 binding, but not all cancer cells or tumors are capable of this adaptive response due to inaccessible enhancers. These chromatin alterations result in gene expression patterns capable of predicting STAT3’s ability to stimulate *ITGB3* expression and represents an epigenetic regulatory motif modulating STAT3 activity. Leveraging this enhanced perspective of STAT3 regulation, we identify 17 additional STAT3-regulated genes with differential chromatin accessibility. Collectively, we refer to this set of 18 genes as the “STAT3-receptive enhancers stratify severity” (*STRESS*) gene signature and demonstrate its ability to stratify survival outcomes of patients with PDAC. We propose that the utility of the *STRESS* signature is such that it predicts how a tumor will dynamically respond to STAT3 signaling resulting from inflammation and oxidative stress and identifies tumors likely to respond to STAT3-targeted therapies. Thus, the signature enables predictive biomarker development for therapeutic decision-making in PDAC.

## RESULTS

### *ITGB3* is a STAT3-regulated stress/inflammatory response gene

**Figure 1.**
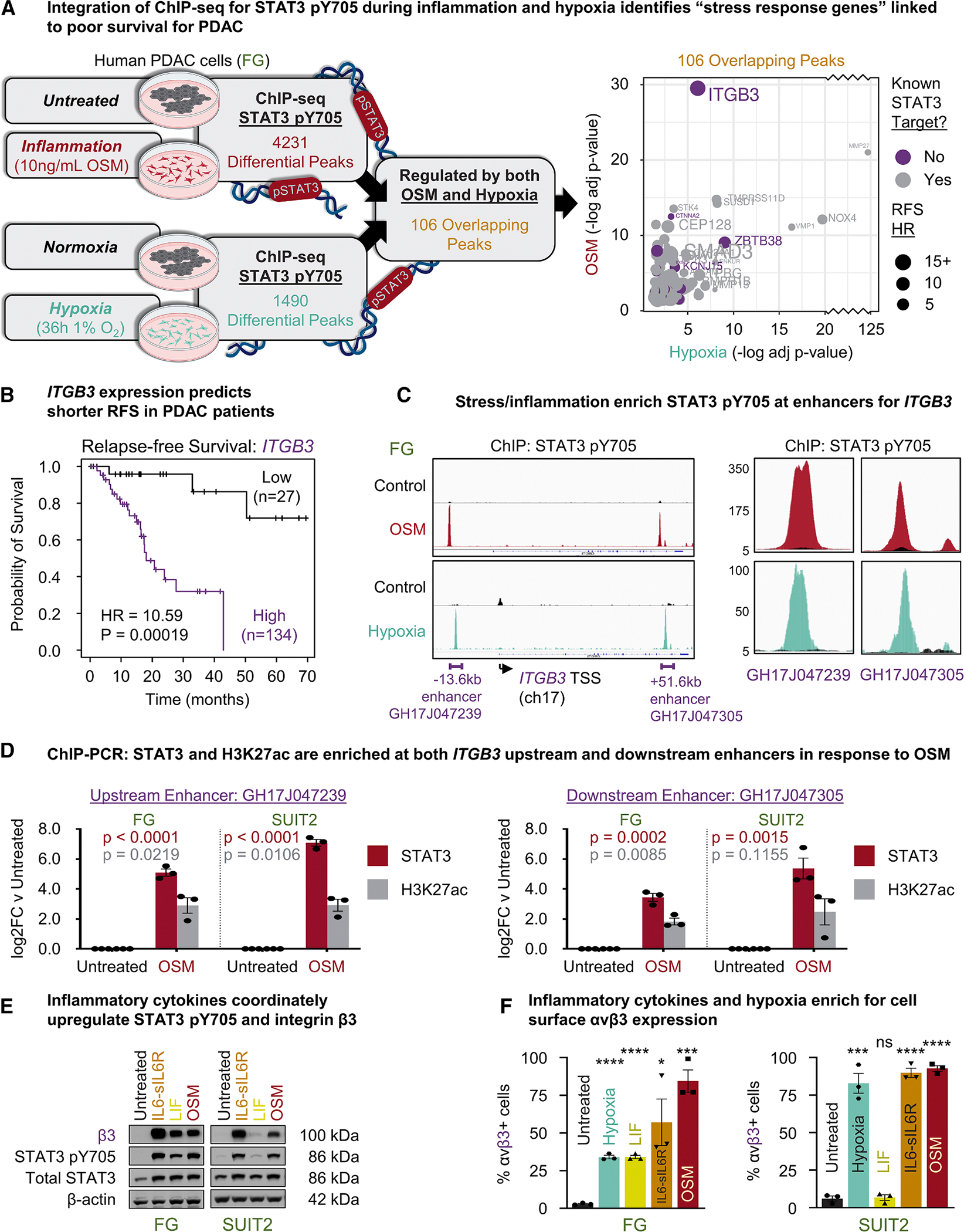
Integrin β3 is a STAT3-regulated stress/inflammatory response gene (A) Schematic (left) depicts parallel ChIP-seq experiments to identify genomic regions to which STAT3-pY705 binds in response to the inflammatory cytokine OSM (10 ng/mL) and hypoxia (1% O_2_) in FG pancreatic cancer cells. Graph (right) shows 106 genes associated with peaks common to both hypoxia and OSM. Hazard ratio (HR); previously unknown STAT3 target (purple); previously known STAT3 target (gray). (B) KM plot shows the probability of relapse-free survival (RFS) in patients with PDAC with high vs. low *ITGB3* expression. (C) ChIP-seq peaks for pSTAT3 at enhancer regions upstream (GH17J047239) and downstream (GH17J047305) of the *ITGB3* transcription start site (TSS) on chromosome 17 (ch17) in response to OSM (red) or hypoxia (teal) vs. control (black). Panels (right) show enlargements of the two enhancers. (D) ChIP-PCR for pSTAT3 and H3K27ac at upstream and downstream enhancers for *ITGB3*. FG and SUIT2 cells were untreated or stimulated with OSM (10 ng/mL) for 72 h. *n* = 3 independent experiments per cell line per condition. Graph displays the mean ± SEM. *p* values reflect Student’s t test vs. untreated. (E) Representative immunoblots for FG and SUIT2 cells treated with vehicle, IL-6-sIL-6R, LIF, or OSM for 72 h. Results represent three independent experiments. (F) Flow cytometry for integrin αvβ3 expression in FG and SUIT2 cells treated with vehicle control/normoxia, IL-6-sIL-6R, LIF, OSM, or hypoxia for 72 h. Graph displays the mean ± SEM for the percent of αvβ3+ cells per group for *n* = 3 independent experiments. ns, not significant, *p < 0.05, **p < 0.01, ***p < 0.001, ****p < 0.0001 by Student’s t test. See also Figure S1 and Table S1.

To identify target genes regulated by STAT3 in response to inflammatory cytokines and hypoxic stress in PDAC, we performed chromatin immunoprecipitation sequencing (ChIP-seq). COLO-357/FG (hereafter “FG”) PDAC cells were cultured in hypoxia (1% O_2_) or normoxia (atmospheric O_2_) and analyzed using ChIP-seq to identify genomic regions with differential STAT3-pY705 DNA binding. In parallel, FG cells were treated with or without oncostatin-M (OSM), an interleukin-6 (IL-6) family member. We identified 4,231 genomic regions (peaks) with differential (*p* ≤ 0.05) STAT3-pY705 binding resulting from OSM treatment and 1,490 resulting from hypoxia. In total, we identified 106 overlapping peaks present in both the hypoxia and OSM datasets that were then annotated using both HOMER^12^ and the GeneHancer database^13^ to identify potential STAT3 target genes. We narrowed the list to 45 genes that were significantly correlated with decreased relapse-free survival (RFS) in patients with PDAC (Figure 1A; Table S1).

Notably, 39 of the 45 (87%) genes were previously reported STAT3 target genes, providing a benchmark validation for our approach. *ITGB3* was the most differentially enriched STAT3 peak in the OSM dataset (adjusted *p* value = 3 × 10^−30^) and the 6^th^ most differential peak in the hypoxia set (adjusted *p* value = 8.4 × 10^−7^). Moreover, *ITGB3* is one of the six novel STAT3 target genes and the most correlated with poor RFS (hazard ratio [HR] = 10.59) (Figure 1B). Importantly, like STAT3, *ITGB3* promotes stress tolerance, tumor initiation, and cancer progression.^9,10,14–16^ Considering the strong correlation between *ITGB3* and poor RFS in patients with PDAC, we characterized the mechanism by which STAT3 regulates *ITGB3* expression and investigated the role of this STAT3/integrin axis in PDAC progression.

Although *in silico* analysis predicted several STAT3 binding motifs in the *ITGB3* promoter region, the ChIP-seq analysis of PDAC cells challenged with inflammation or hypoxia revealed enrichment of STAT3-pY705 binding at two enhancer regions but not at the promoter (Figure 1C). The first enhancer region was located 13.6 kb upstream of the *ITGB3* transcription start site (TSS), corresponding to the GH17J047239 enhancer region described in the GeneHancer database. The second enhancer region (GH17J047305) located within intronic DNA 51.6kb downstream from the *ITGB3* TSS. These findings suggest that STAT3 coordinates the timing and amplitude of *ITGB3* expression, rather than controlling transcription initiation directly. A comprehensive analysis of the ChIP-seq data revealed STAT3 binding was normally distributed among enhancer and promoter regions throughout the genome (Figure S1B). To validate our ChIP-seq data, we performed ChIP, followed by quantitative PCR (ChIP-PCR) in FG and SUIT2 cell lines. In response to OSM stimulation, these cells exhibited increased STAT3-pY705 binding at the previously identified upstream and downstream enhancers. We observed increased histone H3 K27-acetylation, a widely accepted marker for enhancer activity,^17^ and further evidence for STAT3 functioning as an enhancer to stimulate *ITGB3* expression (Figure 1D). Finally, we performed an enriched binding motif analysis among the selected peaks and identified binding motifs associated with AP-1 and STAT transcription factors (Figure S1C).

To validate *ITGB3* as a downstream target of STAT3, we assessed whether various inflammatory cytokines or cellular stress could increase *ITGB3* expression. While the response to cytokines may vary between cell lines that may lack expression of their cognate receptors, the ubiquitous expression of gp130 in most pancreatic cancer cells allows for a response to a complex formed by IL-6 fused to its soluble receptor sIL-6R (IL-6-sIL-6R). Indeed, FG cells showed an induction of both STAT3 Y705 phosphorylation and integrin β3, the protein product of *ITGB3*, in response to OSM, leukemia inhibitory factor (LIF), or IL-6-sIL-6R. In contrast, SUIT2 cells responded only to OSM and IL-6-sIL-6R (Figure 1E), suggesting that this cell line lacks the components required to trigger STAT3 activation in response to LIF. IL-6 family cytokines and hypoxic stress increase *ITGB3* mRNA expression over time (Figure S1D), resulting in increased β3 protein expression (Figure S1E) and presentation of the functional integrin heterodimer (αvβ3) on the cell surface (Figure 1F). Furthermore, the combination of cytokine and hypoxia treatment produced an additive effect on β3 protein expression beyond what was observed with either stimulus alone (Figure S1E).

### *ITGB3* is enriched following chemotherapy and linked to a mesenchymal phenotype

**Figure 2.**
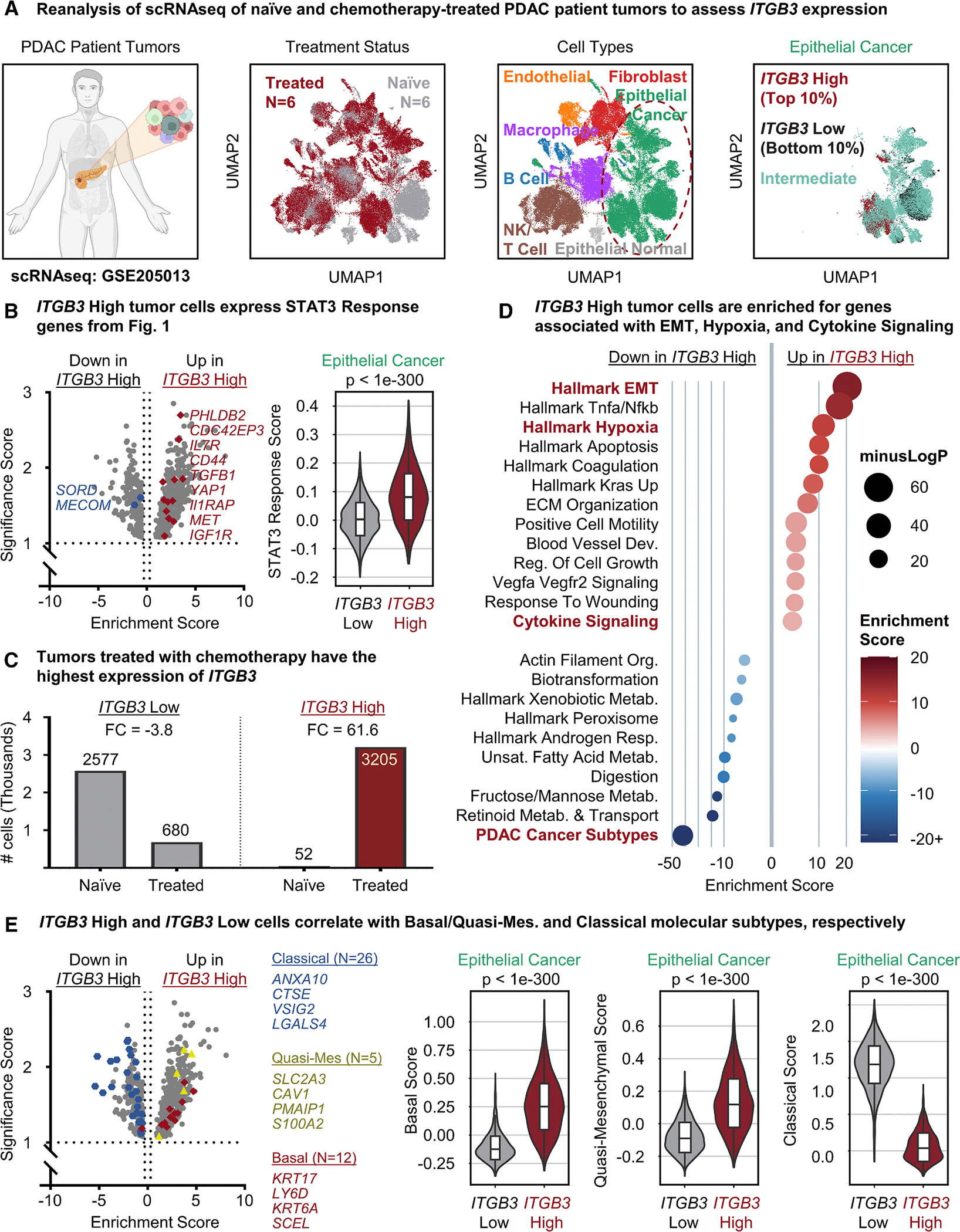
*ITGB3* is enriched following chemotherapy and linked to a mesenchymal phenotype (A) Schematic (panel 1) and UMAP plots of scRNA-seq of *n* = 6 naive (gray) and *n* = 6 chemotherapy-treated (red) patient tumors (panel 2). Cells were annotated by cell type (panel 3) to subset the epithelial cancer cells. The top 10% *ITGB3*-High-expressing (red) and bottom 10% *ITGB3-*Low-expressing (black) expressing cells (panel 4) were assessed for transcriptional differences. (B) DEGs between *ITGB3-*High and *ITGB3-*Low tumor cells (left). Significance score represents negative log10 probability of a gene not being differentially expressed. Enrichment score represents log fold change. Labeled genes are STAT3-regulated genes derived from the ChIP sequencing in Figure 1. Violin plot (right) shows the distribution of STAT3 gene set scores for *ITGB3-*Low and *ITGB3-*High tumor cells. *p* value represents Mann-Whitney U statistic. (C) Plot of the distribution of *ITGB3-*High and *ITGB3-*Low cells between naive and treated patients. Fold change (FC) represents treated vs. naive. (D) Gene set enrichment analysis of DEGs between *ITGB3-*High and *ITGB3-*Low tumor cells. (E) DEGs between *ITGB3-*High and *ITGB3-*Low tumor cells (left). Genes represent classical, quasi-mesenchymal, and basal gene signatures. Violin plots (right) show the distribution of classical, quasi-mesenchymal, and basal gene set scores for *ITGB3-*Low and *ITGB3-*High tumor cells. *p* value represents Mann-Whitney U statistic.

To assess the expression of *ITGB3* in patient tissues, we analyzed previously published^18^ scRNA-seq data from multiple patients with PDAC. Single cells from six patients naive to treatment (naive) and six patients treated with standard-of-care chemotherapy (treated) were annotated by treatment, cell type, and *ITGB3* expression (Figure 2A). Differential expression analysis between epithelial PDAC cancer cells with high *ITGB3* expression (*ITGB3*-High; top 10% of cancer cells) and low *ITGB3* expression (*ITGB3*-Low; bottom 10% of cancer cells) revealed that *ITGB3* highly correlates with the other STAT3 target genes derived from our ChIP-seq data (Figure 2B). *ITGB3*-High cells were overrepresented in treated patients than in naive patients, displaying a 62-fold enrichment (Figure 2C). As both chemotherapy treatment and hypoxia induce oxidative stress,^19,20^ these data strengthen our findings reported in Figure 1 and demonstrate that the ChIP-seq results are highly representative of human pancreatic cancer. Gene ontology analysis showed that differentially expressed genes (DEGs) in *ITGB3*-High cells were significantly enriched for biological processes related to epithelial-to-mesenchymal transition (EMT), hypoxia, and cytokine signaling (Figure 2D). From a clinical perspective, DEGs from *ITGB3*-Low cells were highly correlated with a classical molecular subtype. Conversely, *ITGB3*-High associated with basal (Moffitt^21^) and quasi-mesenchymal (Collisson^22^) molecular subtypes (Figure 2E). These findings not only support our conclusions from the ChIP-seq analysis but also provide clinical relevance for STAT3-mediated *ITGB3* expression in PDAC progression.

### STAT3 is necessary for *ITGB3* expression, which promotes STAT3-mediated tumor initiation/progression

**Figure 3.**
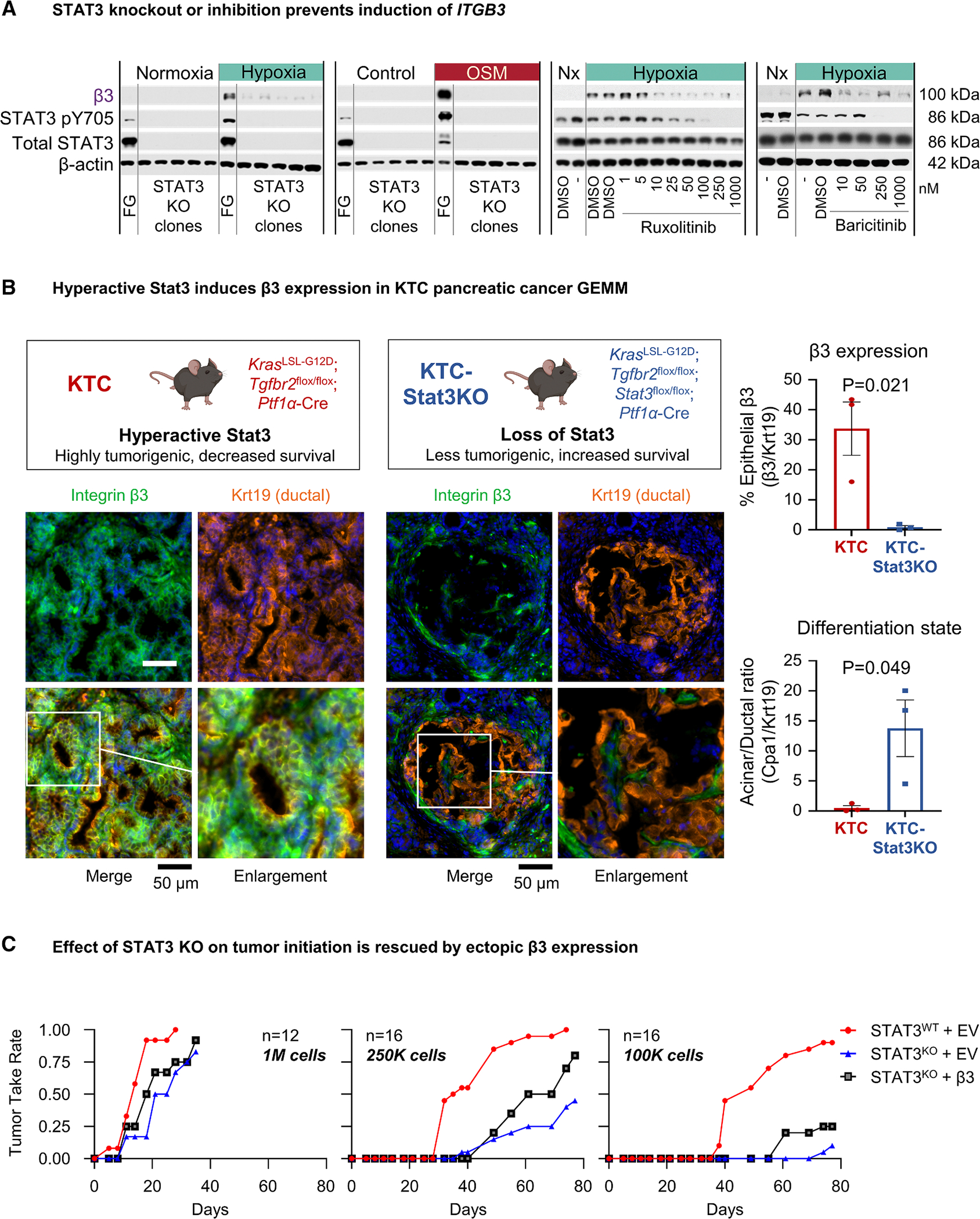
STAT3 is necessary for *ITGB3* expression, which promotes STAT3-mediated tumor initiation/progression (A) FG cells treated with hypoxia, OSM, or respective controls (72 h). Immunoblots show STAT3 (Y705) and integrin β3 for cells with CRISPR KO of STAT3 or pharmacological inhibition of STAT3 upstream regulators using inhibitors. (B) Schematic of KTC vs. KTC-Stat3KO mice. Immunofluorescence staining shows DAPI (blue), integrin β3 (green), and keratin 19 (Krt19, orange) for the representative pancreatic tumor tissue. Scale bar represents 50 μm. Graph (top) depicts the mean ± SEM for the ratio of β3 to Krt19 staining for *n* = 3 tumors per group. Graph (bottom) depicts the degree of differentiation in the pancreas, quantified as the mean ± SEM for the ratio of carboxypeptidase A1 (Cpa1) to Krt19 for *n* = 3 tumors per group. *p* values represent Student’s t test. Additional images and Cpa1 staining are shown in Figure S2D. (C) *In vivo* limiting dilution experiments were performed to assay the tumor-initiating capacities of control FG pancreatic cancer cells (STAT3WT + EV) relative to FG CRISPR STAT3KO with empty vector (STAT3KO + EV) or with ectopic expression of integrin β3 (STAT3KO+β3). Immunocompromised nu/nu mice were injected subcutaneously with varying cell densities. Graphs depict tumor take vs. time for *n* = 12–20 tumors per group. See also Figure S2.

To investigate whether cytokines and cellular stress stimulate *ITGB3* expression in a STAT3-dependent manner, we developed *STAT3* CRISPR knockout (KO) clones in FG pancreatic cancer cells. Compared with STAT3^WT^ control cells, STAT3^KO^ cells did not show an induction of integrin β3 in response to either hypoxia or OSM (Figure 3A). Similarly, cells transiently expressing *STAT3* siRNA did not gain integrin β3 expression in response to hypoxia (Figure S2A). Furthermore, the inhibition of STAT3’s upstream regulators, JAK1/2, with the Food and Drug Administration (FDA)-approved inhibitors ruxolitinib or baricitinib prevented hypoxia- and LIF-induced β3 expression (Figures 3A and S2B). Thus, genetic silencing of STAT3 or pharmacological inhibition of its upstream kinases prevents pancreatic cancer cells from gaining integrin β3 expression in response to cytokines or stress *in vitro*.

We next asked if STAT3 is necessary for β3 expression on tumor cells *in vivo* using a spontaneous mouse model of pancreatic cancer. Laklai and colleagues^23^ previously demonstrated that in the context of mutant *Kras*, a loss of Tgf-β receptor 2 signaling resulted in elevated Stat3 signaling and tumor progression, while epithelial *Stat3* ablation attenuated tumor progression and prolonged survival. We profiled pancreatic tissues from KTC mice, which develop hyperactive Stat3 signaling due to the pancreas-specific expression of *Kras-G12D* and homozygous loss of *Tgfbr2* (*Kras*^LSL-G12D^/*Tgfbr2*^flox/flox^/*Ptf1α*-Cre), and KTC-Stat3KO mice harboring a conditional *Stat3* knockout (*Kras*^LSL−G12D^/*Tgfbr2*^flox/flox^/*Stat3*^flox/flox^/*Ptf1α*-Cre). As reported previously for this model,^23^ *Stat3* knockout resulted in a less severe pathology accompanied by maintenance of carboxypeptidase A1 (Cpa1)-positive acinar cells and fewer keratin-19 (Krt19)-positive ductal lesions (Figures 3B and S2C). Consistent with Stat3-mediated control of *Itgb3* expression, the integrin β3 protein was abundantly expressed on Krt19-positive ductal lesions in the pancreas of KTC mice but absent on epithelial lesions in KTC-Stat3KO tissues. Although β3 expression is lost on the cell membrane of epithelial cells in KTC-Stat3KO mice, it is still observed in fibrovascular cells, further validating the conclusion that Stat3 mediates β3 expression since *Stat3* is only ablated in epithelial cells expressing *Ptf1a* in this model (Figure 3B). Compared with KTC tumors with hyperactive Stat3 and abundant integrin β3 expression, KTC-Stat3KO tumors that lack β3 expression were reported to be far less aggressive,^23^ supporting a role for the STAT3/β3 axis in tumor progression.

To further interrogate the role of *ITGB3* as a critical downstream effector of STAT3-mediated human tumor initiation *in vivo*, we performed a limiting dilution assay to compare the ability of FG STAT3^WT^ vs. FG STAT3^KO^ cells (Figure S2D) to establish tumors in nude mice. As cell number decreased and the requirement for resilience under pressure increased, STAT3^KO^ severely attenuated tumor-initiating capacity (i.e., tumor take rate). Ectopic expression of integrin β3 in STAT3^KO^ cells partially restored tumor initiation, bypassing the requirement for STAT3 (Figure 3C). These findings indicate that a loss of STAT3 signaling compromises *ITGB3* induction, underscoring the essential role of the STAT3/β3 axis in pancreatic cancer initiation.

### Ability to gain integrin β3 enhances tumor initiation

**Figure 4.**
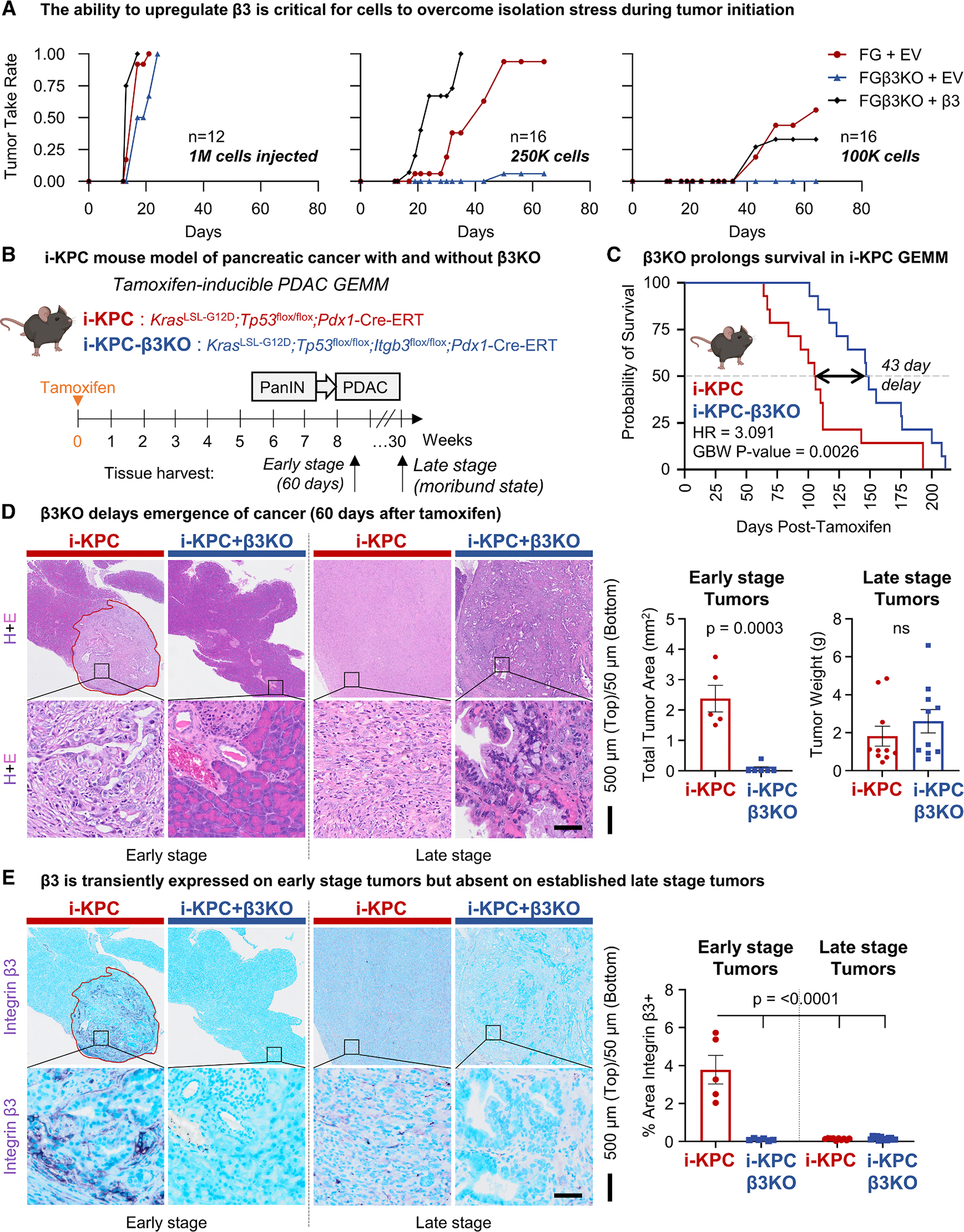
Ability to gain integrin β3 enhances tumor initiation (A) Limiting dilution tumor initiation assay of control FG pancreatic cancer cells (FG + EV) vs. FG CRISPR β3KO cells with empty vector (FG β3KO + EV) or ectopic β3 (FG β3KO+β3). Immunocompromised nu/nu mice were injected subcutaneously with 1 × 10^6^, 2.5 × 10^5^, or 1 × 10^5^ cells per injection. Graphs depict tumor take vs. time for *n* = 12–16 tumors per group. (B) Schematic depicts 6-week-old tamoxifen-inducible KPC (i-KPC; *Kras*^LSL−G12D^/*Tp53*^flox/flox^/*Pdx1*-Cre^ER^) mice crossed with *Itgb3*^flox/flox^ mice to establish tamoxifen-inducible i-KPC-β3KO (*Kras*^LSL−G12D^/*Tp53*^flox/flox^/*Itgb3*^flox/flox^/*Pdx1*-Cre^ER^) mice. (C) Probability of survival vs. time following tamoxifen injection for *n* = 14 mice per group. The HR and *p* value reflect KM analysis. (D) Serial sections of early-stage (60-day) pancreatic tissues (*n* = 5 i-KPC, *n* = 6 i-KPC-β3KO) were stained using hematoxylin and eosin (H&E) and quantified for the total tumor area (left graph; mean ± SEM). Late-stage tumors (*n* = 14 i-KPC, *n* = 14 i-KPC-β3KO) were measured by tumor weight (right graph; mean ± SEM). Tumor area is outlined in red. Representative images for each group are shown. Scale bars represent 500 μm (top) and 50 μm (bottom). (E) Early-stage and late-stage tumors were stained for integrin β3. Positive staining is graphed as the mean ± SEM percent of the total tumor area on whole-tissue sections. *p* values represent one-way ANOVA with Tukey’s multiple comparisons. Scale bars represent 500 μm (top) and 50 μm (bottom). See also Figure S3 and Table S2.

To further establish the role of integrin β3 in PDAC initiation, we developed *ITGB3* CRISPR KO FG cells (FGβ3^KO^). Notably, unstimulated or unstressed FG cells had no detectable cell surface integrin αvβ3. While wild-type (WT) controls upregulated αvβ3 in response to OSM, FGβ3^KO^ cells did not (Figure S3A). We then assessed the tumor-initiating capacity of FGβ3^KO^ relative to FG cells in a limiting dilution assay, as described above. While no difference in tumor take was observed between the FG and FGβ3^KO^ conditions when 1 × 10^6^ cells were implanted into mice, there was a dramatic impairment in the tumor-initiating capacity of FGβ3^KO^ cells when fewer cells were implanted. This deficit was rescued by ectopic expression of integrin β3 (FGβ3^KO^ + β3) (Figure 4A). Thus, the ability to upregulate integrin β3 is critical for a cell to overcome isolation stress during tumor initiation.

To determine the impact of β3 acquisition on the spontaneous development and progression of pancreatic cancer, we crossed mice from the previously developed^24^ triple mutant model of tamoxifen-inducible PDAC (i-KPC; *Kras*^LSL-G12D^/*Tp53*^flox/flox^/*Pdx1*-Cre^ER^), with mice expressing a conditional homozygous loss of *Itgb3* (i-KPC-β3KO; *Kras*^LSL−G12D^/*Tp53*^flox/flox^/*Itgb3*^flox/flox^/*Pdx1*-Cre^ER^) (Figure 4B). Adult, 6-week-old i-KPC mice treated with tamoxifen developed large pancreatic tumors and became moribund at a median of 15 weeks. In contrast, deletion of epithelial integrin β3 prolonged survival by an additional 6 weeks (140%) in this highly aggressive model of pancreatic cancer (Figure 4C; Table S2). Interestingly, once tumors overcame the deficit conferred through a loss of integrin β3 during the initiation phase of the cancer, there was no difference in the mortality rate or end-stage tumor size (Figures 4C and 4D), suggesting that alternative pathways can eventually compensate for the inability to gain β3 during tumor initiation and early lesion formation. Tumors harvested 60 days (early stage) after tamoxifen treatment revealed that an inability to upregulate β3 delays tumor initiation. The i-KPC-β3KO pancreatic tissue appeared healthy, with normal acinar cells, ducts, and islet cells, while the i-KPC pancreatic tissue contained the expected proportions of healthy tissues, low-grade PanINs, high-grade PanINs, and small tumors (Figures 4D and S3B). At this early stage, high expression levels of integrin β3 were observed in Krt19-positive metaplastic regions but not on Alcian blue-positive PanIN structures (Figures 4E and S3B). Surprisingly, in late-stage tumors harvested upon observing moribund signs, β3 expression was largely undetectable (Figures 4E). Together, these results support the conclusion that β3 is transiently upregulated to facilitate tumor initiation but gets downregulated once a tumor is established.

### *ITGB3* expression promotes progression from a classical to basal subtype in advanced tumors

**Figure 5.**
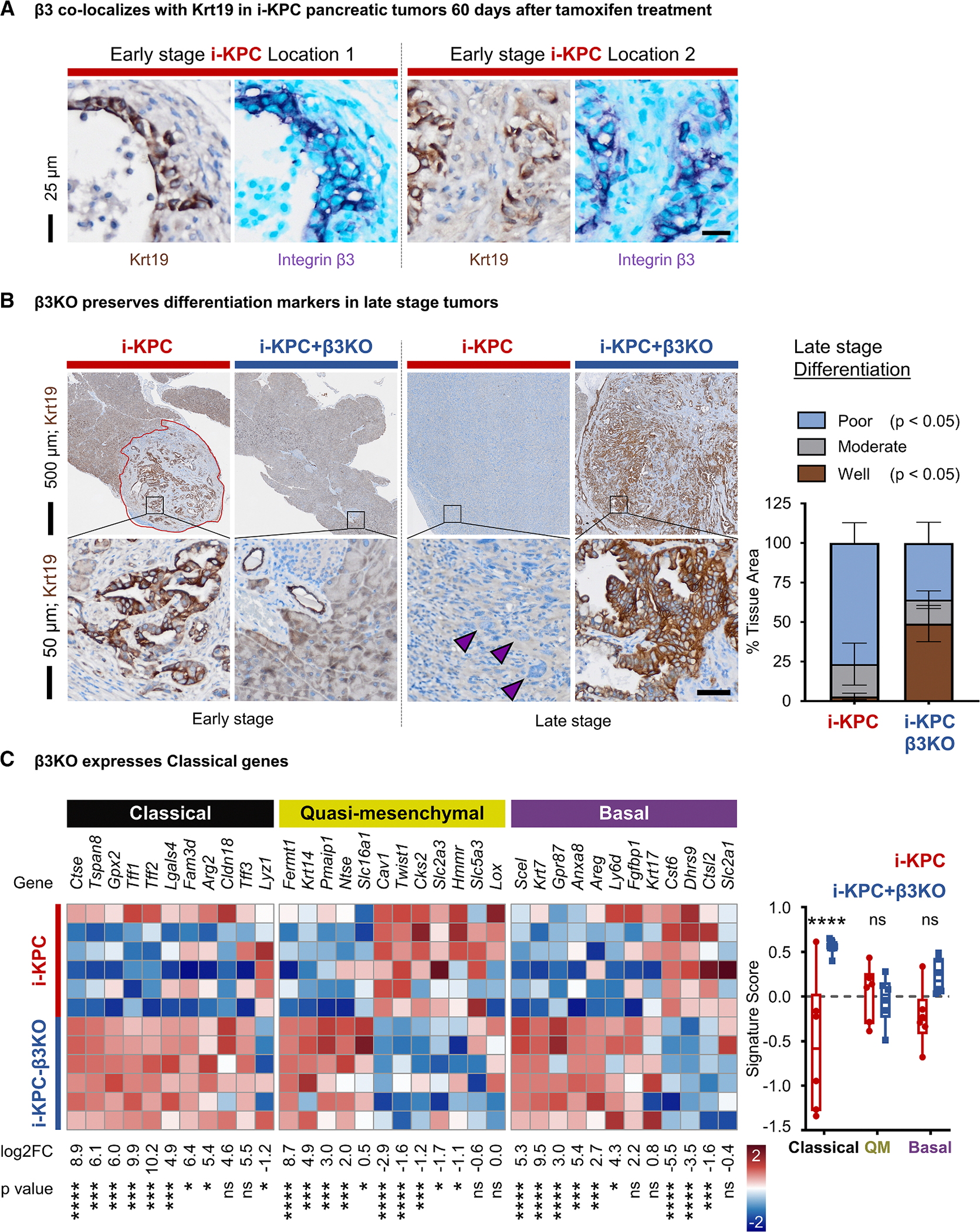
*ITGB3* expression promotes progression from a classical to basal subtype in advanced tumors (A) Representative immunohistochemistry demonstrating co-localization of β3 (purple) with Krt19 (brown) in early-stage i-KPC tumors. Scale bars represent 25 μm. (B) Representative Krt19 immunostaining of end-stage tumors. Scale bars represent 500 μm (top) and 50 μm (bottom). Krt19 expression classified by blinded observers into poorly, moderately, and well-differentiated categories. Graph depicts the mean ± SEM for the percent tissue area for each category of differentiation for 10 i-KPC tumors and 13 i-KPC-β3KO tumors. *p* values represent two-way ANOVA with Šidák’s multiple comparisons. (C) RNA quantified using RT-qPCR on flash-frozen late-stage tumors (*n* = 6 i-KPC, *n* = 6 i-KPC-β3KO). Heatmap (left), upregulation (red), and downregulation (blue) depicting classical, quasi-mesenchymal, and basal gene sets plotted as the *Z* score (per gene) of log2 FC. Graph (right) depicts summarized gene set score for subtypes calculated as the mean of the *Z* scores for genes in each subtype. ns, not significant, *p < 0.05, **p < 0.01, ***p < 0.001, ****p < 0.0001 by Student’s t test (heatmap; left) and unpaired t test with single pooled variance (graph; right).

Although integrin β3 expression is transiently upregulated on cancer cells during tumor initiation and easily detectable at the early-stage time point (Figure 5A), the effects of β3 expression remain evident even after β3 expression is lost. As judged by two blinded observers, late-stage tumors from i-KPC-β3KO mice showed a higher percentage of tumor areas that were scored as moderately to well differentiated relative to late-stage tumor areas from i-KPC mice (Figure 5B; Table S2). These moderate and well-differentiated tumors displayed a classical ductal morphology with glandular features and were often more associated with a classical molecular phenotype. Conversely, i-KPC mice were primarily observed to be poorly differentiated (i.e., not making glands or showing sarcomatous features), which have previously been reported to associate with a more aggressive phenotype for this pancreatic cancer model^24^ (Figure 5B). When assessed for gene expression patterns associated with basal, quasi-mesenchymal, and classical molecular subtypes, late-stage i-KPC-β3KO tumors were enriched for classical subtype genes relative to i-KPC tumors (Figure 5C). These expression data are consistent with our analysis of scRNA-seq data from human patient samples, which demonstrated that PDAC cells with low *ITGB3* expression maintained a classical molecular subtype (Figure 2E). These findings suggest that β3 expression during early-stage tumor development promotes a shift away from the classical phenotype to a more basal/quasi-mesenchymal phenotype at later stages, despite the downregulation of β3 expression as tumors become more established.

### Chromatin accessibility for STAT3 binding dictates the ability to express integrin β3

**Figure 6.**
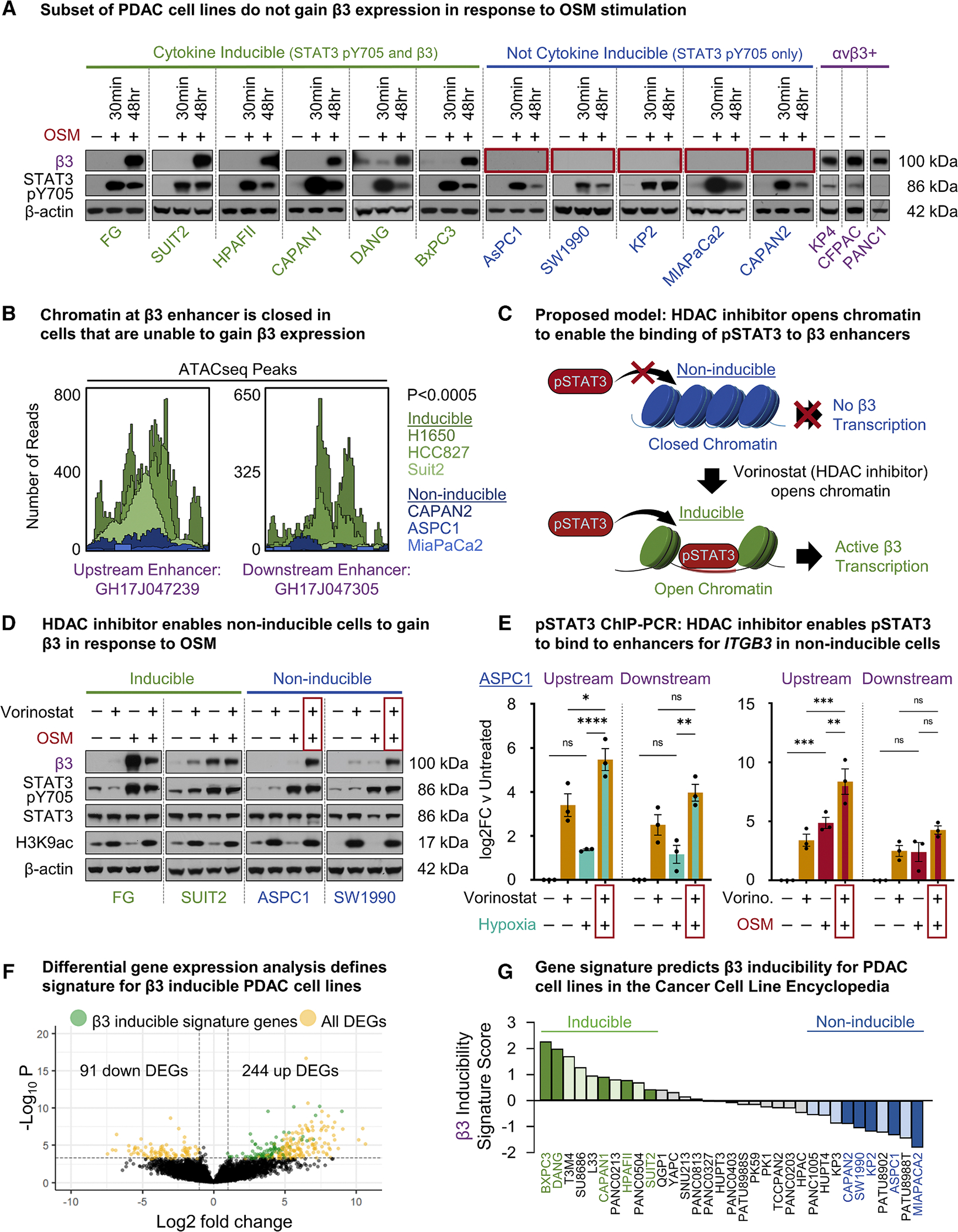
Chromatin accessibility for STAT3 binding dictates the ability to express integrin β3 (A) PDAC lines were treated with vehicle or 10 ng/mL OSM for either 30 min or 48 h. Immunoblots for integrin β3 and STAT3-pY705. (B) Depiction of ATAC-seq peaks at the GH17J047239 and GH17J047305 enhancers for PDAC lines. Inducible cells, green; non-inducible cells, blue. *p* value indicates significance following multiple testing correction (adjusted *p*) of the log2 fold difference for the mean peak area of reads in each enhancer region computed for *n* = 3 cell lines per group (inducible vs. non-inducible). (C) Model whereby chromatin accessibility at *ITGB3* enhancers controls STAT3 binding to induce β3 expression. Vorinostat promotes chromatin opening to enable STAT3 binding. (D) Inducible vs. non-inducible cells were untreated or treated with OSM (10 ng/mL) and vorinostat (5 μM) alone or in combination and then immunoblotted for β3, STAT3-pY705, total STAT3, H3K9ac, and β-actin. Representative of *n* = 3 independent experiments per cell line per condition. (E) A non-inducible cell (ASPC1) was untreated or treated with hypoxia (1% oxygen) and vorinostat (5 μM) alone or in combination (left). Alternatively, cells were untreated or treated with OSM (10 ng/mL) and vorinostat (5 μM) alone or in combination (right). pSTAT3 binding at enhancers for *ITGB3* was measured using ChIP-PCR, plotted as the mean ± SEM log2 FC relative to untreated for *n* = 3 independent experiments per condition. ns, not significant, *p < 0.05, **p < 0.01, ***p < 0.001, ****p < 0.0001 by ordinary one-way ANOVA with Tukey’s multiple comparisons. (F) Volcano plot illustrates DEGs between five inducible and five non-inducible lines. Significant DEGs (yellow) have log2 FC > 1 or < −1 and adjusted *p* value < 0.05. Genes comprising the β3-inducible signature (green) are defined as upregulated with normalized gene count standard deviations of <2. (G) CCLE PDAC lines were assigned β3-inducible signature scores and converted to *Z* scores and ranked. Dark green and dark blue bars represent cell lines with known inducible and non-inducible (respectively) phenotypes. Light green and light blue bars represent cell lines predicted to have inducible or non-inducible phenotypes, respectively. See also Figures S4 and S5.

We expanded our studies to include 14 PDAC cell lines. Surprisingly, the cell lines on this panel were categorized into three phenotypes with respect to integrin β3 expression: (1) cell lines with endogenous expression at baseline, (2) cell lines that induced β3 in response to cytokine or hypoxia (inducible), and (3) cell lines that could not induce β3 (non-inducible) (Figure 6A). Although all β3-negative cell lines gained STAT3-pY705 in response to cytokine treatment, only FG, SUIT2, HPAFII, CAPAN1, DANG, and BxPC3 cells induced integrin β3 expression, while CAPAN2, ASPC1, MIAPACA2, SW1990, and KP2 cells did not.

Because responsiveness to a given cytokine depends on the expression of cognate cytokine receptors that show heterogeneous expression between cell lines, a smaller panel of cell lines was screened to determine if the response to OSM would extend to additional cytokines that activate STAT3 (Figure S4A). Consistent with the response to OSM, all cytokines that increased STAT3-pY705 also increased β3 expression in inducible cells but not in non-inducible cells. The notable exception was SW1990 cells, which gained β3 expression in response to IL-6-sIL-6R but not OSM or IL-27. TGF-β1, a known regulator of β3 expression,^25^ induced β3 expression independent of STAT3 in inducible cells but not in non-inducible cells.

In addition to its role as a transcription factor, STAT3 can impact gene expression through its enhancer activity and recruitment of chromatin remodelers.^26,27^ We postulated that differences in β3 inducibility may arise from differences in chromatin accessibility. To explore this, we accessed publicly available ATAC-seq data for a panel of human cancer cell lines that we empirically validated to be inducible or non-inducible. Inducible cells have open chromatin at both of the two previously described upstream (GH17J047239) and downstream (GH17J047305) STAT3 enhancer regions for *ITGB3*. Conversely, Non-inducible cells exhibited closed chromatin at these same enhancer sites (Figures 6B, S4B, and S4C), suggesting chromatin must be in an open/accessible state for STAT3 binding to stimulate β3 transcription (Figure 6C).

To test whether an open/accessible chromatin state is required for the induction of β3, we treated both β3 Inducible and Non-inducible PDAC cell lines with the histone deacetylase (HDAC) inhibitor vorinostat, an FDA-approved “epigenetic drug” that modulates histone acetylation to promote chromatin opening. Vorinostat enabled Non-inducible cells to gain β3 in response to OSM or hypoxia, where vorinostat, OSM, or hypoxia alone did not, suggesting that both open chromatin and active STAT3 signaling are required for β3 expression (Figures 6D and S5A).

Next, we investigated if forcing chromatin into an open state enables STAT3 to bind at β3 enhancers where it otherwise could not. We performed ChIP-PCR in Non-inducible cells treated with individual or combined treatments as described above. While OSM treatment alone partially increased STAT3 binding at β3 enhancers, the combination of vorinostat with OSM stimulated significantly more STAT3 binding compared to either treatment alone. We observed similar results in cells treated with the combination of vorinostat and hypoxia (Figures 6E and S5B). Importantly, because OSM or hypoxia treatment alone was insufficient to stimulate β3 expression in Non-inducible cells despite increased binding, these results suggest that a minimum threshold of STAT3 binding is required to induce β3 expression.

Since changes in chromatin accessibility result in wide-scale gene expression changes,^28^ we considered if differences in baseline gene expression could be exploited to discern β3 inducible from non-inducible phenotypes for PDAC cell lines and tumors with unknown β3 inducibility phenotype. We used DepMap^29^ to acquire RNA-seq gene expression data for 40 PDAC lines in the Cancer Cell Line Encyclopedia (CCLE). This collection included gene expression data for 10 PDAC cell lines that we empirically validated to be β3-inducible (*N* = 5) or non-inducible (*N* = 5) (Figure 6A). We identified DEGs between the inducible and non-inducible cell lines, of which 93 genes upregulated in inducible cells were selected to develop a gene signature that would reflect a cell line’s ability to induce β3 (Figures 6F and S5C). The β3-inducible gene signature was then applied using rank-based signature scoring to the remaining PDAC lines of unknown inducibility to generate predictions for the cell’s β3 inducibility (Figure 6G). Unsupervised principal-component analysis showed that confirmed and predicted inducible cell lines had gene expression patterns that were distinct from those of confirmed and predicted non-inducible cell lines (Figure S5D). Cell lines with intermediate scores clustered in between these two phenotypes. Notably, L3.3 (L33) is a metastatic variant derived from COLO-357, the same parent cell line for the β3-inducible FG variant utilized in this study.^30^ While L33 expression data were not utilized to generate the β3-inducible gene signature, its inducibility status was accurately predicted as a β3-inducible cell line. Thus, alterations in chromatin accessibility regulate gene expression patterns that portend a β3-inducible phenotype.

To demonstrate a physiological significance for β3-inducible signature scores in PDAC cell lines, we leveraged the MetMap scores that characterize an individual cell line’s metastatic potential for distinct organs.^31^ We first segregated PDAC cell lines as metastasis derived or primary derived. Most cell lines derived from metastatic sites reported a high metastatic potential, whereas cell lines derived from primary tumors showed a range of MetMap scores. For cell lines derived from primary sites, there was a significant positive correlation between the MetMap score and the β3 inducibility signature score (*p* = 0.02) (Figure S5E). These findings reinforce the correlation observed between an EMT gene expression pattern and cancer cells expressing high *ITGB3* expression in patients with PDAC.

### Subset of STAT3 targets (*STRESS* gene set) reflects the critical role for STAT3 during tumor initiation and progression of PDAC

**Figure 7.**
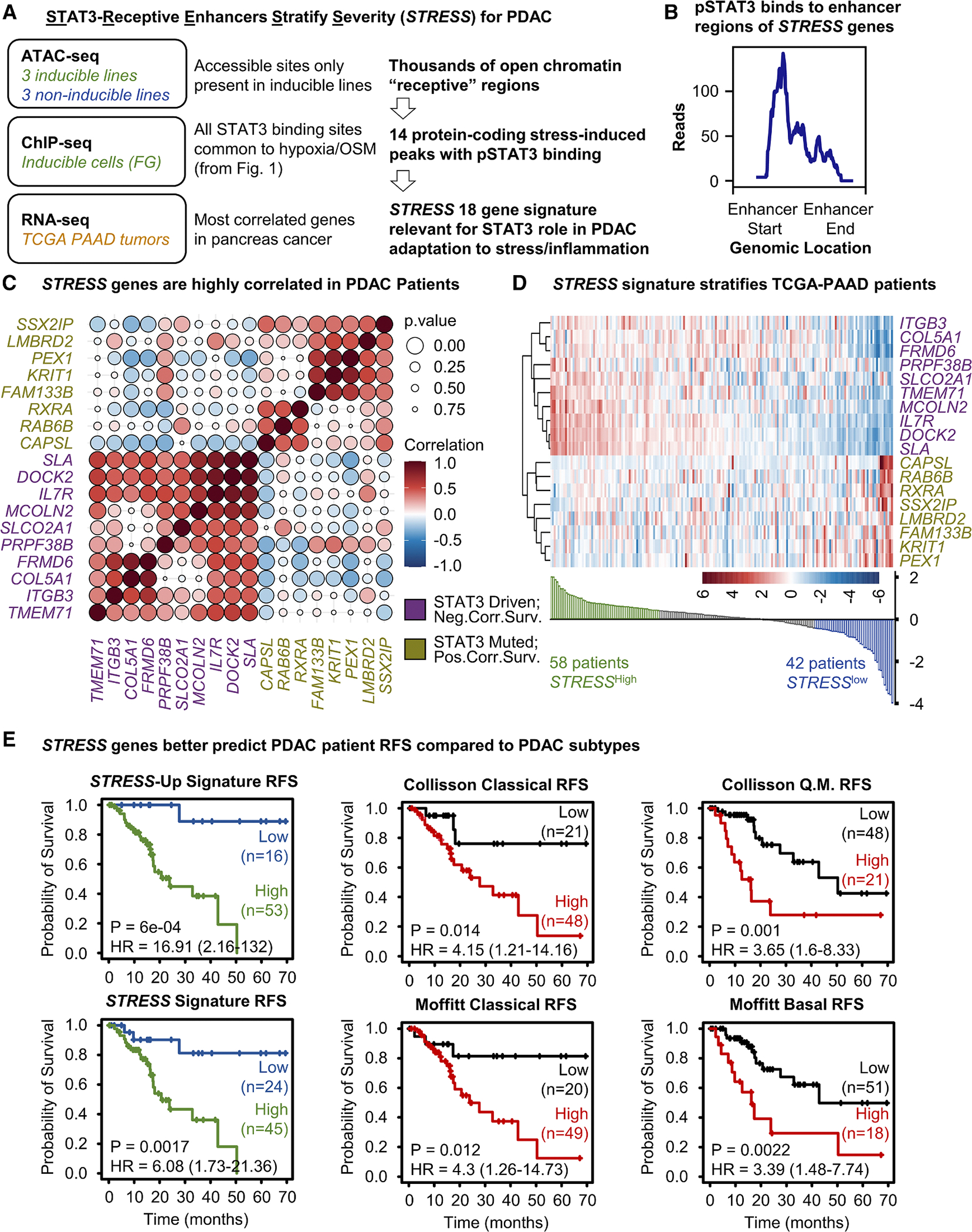
Subset of STAT3 targets (*STRESS* gene set) reflects the critical role of STAT3 during tumor initiation and progression of PDAC (A) Schematic summarizes steps used to generate the 18-gene *STRESS* signature. (B) Graph shows the number of genomic read pile-ups for STAT3-pY705 binding at enhancers. (C) Correlation matrix depicts the co-expression Pearson correlation coefficient (according to the color map) and corresponding *p* value (size of each circle) for the 18 genes included in the *STRESS* signature for patients in The Cancer Genome Atlas (TCGA) PAAD dataset. Genes indicated in purple are those that are likely driven by STAT3 and whose high expressions are negatively correlated with survival as a consequence. Genes in gold are those that are likely repressed following STAT3 binding to enhancers and whose high expressions are positively correlated with survival. (D) Upper panel shows unbiased clustering of the 18-genes *STRESS* signature for TCGA-PAAD dataset. Lower panel depicts value of the computed *STRESS* signature score. (E) KM plot of RFS in patients with PDAC according to the *STRESS* or *STRESS*-*Up* signatures compared with Collisson and Moffitt signatures. See also Figures S6 and S7 and Table S3.

Having demonstrated that β3 is critical for PDAC initiation and progression, that its expression is dictated by chromatin accessibility of its STAT3 enhancers, and that not all cells are capable of upregulating its expression, we aimed to identify additional genes relevant to human PDAC that are regulated similarly to β3, with the goal of improving patient stratification and predicting survival outcomes. As summarized in Figure 7A, we cross-referenced ATAC-seq data (Figure 6) with STAT3-pY705 ChIP-seq data (Figure 1) to identify accessible sites present only in inducible lines that are also regions at which STAT3 binds in response to OSM and hypoxia. This initial analysis yielded 14 conserved regions representing receptive enhancers for stress-induced STAT3-pY705 binding (Figure 7B). According to the GeneHancer database, these 14 regions are predicted to function as enhancers for 54 putative target genes (Figure S6A). We hypothesized that genes regulated by the same mechanism should be highly correlated in RNA expression data from The Cancer Genome Atlas Pancreatic Ductal Adenocarcinoma (TCGA-PAAD) cohort. Of the 54 putative target genes, 18 genes with the strongest co-expression correlations were selected (Figures 7C and S6B). Among these, 10 genes were negatively correlated with survival and were driven by STAT3, while the remaining 8 genes were positively correlated with survival and repressed by STAT3 (Figure 7C). We referred to the complete set of 18 genes as the “STAT3-receptive enhancers stratify severity” (*STRESS*) gene signature and the 10 positively correlated genes as the *STRESS*-*Up* signature. To validate our findings, we assessed mRNA expression levels of the *STRESS-Up* genes in inducible and non-inducible PDAC cell lines following OSM stimulation. Six of the 10 genes expressed were upregulated in the inducible, but not in the non-inducible, cell line, while one gene did not respond as expected (Figure S6C). The remaining three were not detected in either group.

For the TCGA-PAAD patient cohort, the *STRESS* signature stratified patients into high vs. low groups (Figure 7D). Using the Kaplan-Meier (KM) plotter^32^ with “auto select best cutoff,” we found that high expression of the *STRESS* signature was associated with significantly higher HR, reflecting a shorter RFS (HR = 6.08, *p* = 0.0017) while stratification using the *STRESS-Up* signature was even more profound (HR = 16.91, *p* = 0.0006) (Figure 7E). Interestingly, the *STRESS* and *STRESS-Up* signatures were more powerful to stratify patients with poor RFS than previously reported molecular subtypes of pancreatic cancer, including Collisson’s classical/quasi-mesenchymal subtypes and Moffitt’s classical/basal subtypes^33,34^ (Figure 7E). Correlation analyses showed that a high *STRESS* signature does not identify the same patients as the Collisson or Moffitt signatures (Figures S6D and S6E), and none of the genes included in the *STRESS* signature were represented in either the Collisson or Moffitt signatures (Figure S7A; Table S3). In contrast, the *STRESS* signature had a strong positive correlation to the cell line-derived β3-inducible signature in patients in the TCGA-PAAD cohort (Figure S7B), despite having no overlap in genes (Figure S7C; Table S3), reinforcing the idea that a cell can activate specific gene expression programs only if they possess the requisite chromatin-accessible states at specific enhancers to which STAT3 binds. It is also interesting to note that while the *STRESS* and Collisson/Moffitt signatures were strongly associated with poor RFS, their association with poor overall survival in the TCGA-PAAD dataset was less strong (Figure S7D). This observation echoes the impact of β3 knockout in the KPC mouse model of pancreatic cancer, in which there was a significant delay in tumor initiation yet no difference in primary tumor size or overall mortality rate (Figure 4). Together, our findings support the identification of a subset of STAT3 target genes useful in identifying tumors with STAT3 dependency and the ability to stratify survival outcomes for patients with pancreatic cancer.

## DISCUSSION

While STAT3 has been linked to the initiation and progression of various cancers, including pancreatic cancer, its ability to function as a transcription factor for thousands of target genes is known to vary widely among different cell lineages, disease states, or microenvironments.^35–38^ We reasoned that a more detailed understanding of how PDAC activation of STAT3 in response to stress and inflammation contributes to the malignancy of pancreatic cancer might uncover critical genes that regulate the initiation and progression of this highly aggressive cancer. A goal of this study was to learn how pancreatic cancer cells hijack specific functions of STAT3 that allow them to respond to stress and inflammation encountered within the tumor microenvironment.

Accordingly, we identified genes upregulated by STAT3 in pancreatic cancer cells exposed to hypoxia and inflammatory cytokines, reasoning that their intersection would represent a fundamental role for STAT3 in adapting to the effects of cellular stress or inflammatory signals. We narrowed the list of relevant STAT3 targets by their significant inverse correlation with RFS in patients with PDAC. We chose to validate *ITGB3*, as it was identified as the top previously unknown STAT3 target, and its expression drives the presentation of integrin αvβ3 on the cell surface. Importantly, integrin αvβ3’s functional activities overlap with many of the pro-tumor functions of STAT3^9–11,38^ since both are drivers of cancer progression and tumor stem cell behavior. Both STAT3 and αvβ3 enable cancer cells to overcome the effects of stress by activating pathways that promote EMT, drug resistance, and metabolic flexibility.^9–11,14–16^ We recently reported that challenging lung cancer cells with nutrient stress upregulated *ITGB3* expression to drive chronic activation of the stress sensor AMP-activated protein kinase (AMPK), shifting cancer cell metabolism toward oxidative phosphorylation and facilitating survival in the nutrient stressed environment.^15^ Here, we demonstrate that STAT3’s ability to promote tumor initiation can be attributed, in part, to its role in facilitating the upregulation of *ITGB3* expression in pancreatic cancer cells, since ectopic expression of a single STAT3 target gene (i.e., *ITGB3*) could restore some of the tumor-initiating ability of STAT3-KO cells. Using a series of *in vitro* and *in vivo* models, we show that STAT3 is required for pancreatic cancer cells to upregulate integrin β3 expression in response to hypoxia or cytokine stimulation, which enhances a cell’s ability to initiate a tumor *in vivo*.

Investigating how STAT3 regulates β3 expression in pancreatic cancer cells revealed a striking dichotomy, since only certain cell lines lacking endogenous β3 expression could upregulate its expression. We attribute this distinction to chromatin accessibility at *ITGB3* enhancer regions, creating a “receptive” location for STAT3 binding. Comparison of baseline gene expression profiles among cell lines that could vs. could not induce *ITGB3* expression allowed us to identify a gene expression pattern predicting the potential for a cell to induce *ITGB3* expression. The ability to induce β3 is necessary for a cell to initiate tumors at primary or metastatic sites, as evidenced by our *in vivo* data and supported by informatics analysis showing that non-inducible PDAC cell lines have a lower metastatic potential and *ITGB3*-High PDAC tumor cells in patients associated with EMT and the basal molecular phenotypes. Notably, integrin β3, EMT, and basal molecular phenotypes have been reported to drive metastatic progression.^10,39–45^ We then extended this concept to create an 18-gene signature (*STRESS*) to identify patient tumors capable of STAT3-mediated adaptation to stress/inflammation, as defined by STAT3-pY705 binding to differentially accessible enhancers regulating target genes, which are also highly correlated with poor survival in the TCGA pancreatic cancer dataset. Within this signature were 10 *STRESS-Up* genes that were upregulated and showed an even stronger correlation with poor survival in patients with PDAC.

Our work highlights new opportunities to target STAT3 or its effectors in molecularly defined subpopulations of patients who are more likely to respond based on their expression of *STRESS* signature genes. Despite biological evidence supporting STAT3’s role in a variety of cancers, drugs targeting the JAK/STAT3 pathway have yet to produce significant improvements in clinical outcomes, including several clinical trials that included patients with pancreatic cancer.^38,46,47^ The lack of success of JAK/STAT3 inhibitors may be due in part to the ability of such therapeutics to achieve effective STAT3 blockade in patients or due to dose-limiting toxicities from off-target adverse events.^48^ It is also complicated to unravel the mechanisms of action for STAT3 blockade, considering STAT3’s contributions to multiple cell types within the tumor microenvironment. For example, the JAK/STAT3 inhibitor ruxolitinib in combination with inhibitors targeting mitogen-activated protein kinase (MEK) and programmed cell death protein 1 (PD-1) is currently being tested in a phase 1 trial for patients with metastatic PDAC (NCT05440942), a cocktail intended to overcome immunotherapy resistance by targeting cancer-associated fibroblasts and reprogramming tumor-associated macrophages.^49^ Similarly, direct integrin β3 antagonists have failed to improve patient outcomes.^50,51^ This could be because αvβ3, even in the unligated state, plays a significant yet transient role during tumor initiation before the cancer is diagnosed and possibly much later in disease progression as tumor cell drug resistance occurs.

The *STRESS* gene signature represents genes inducible by stressors or inflammatory mediators in the tumor microenvironment and are notably distinct from previously identified PDAC molecular subtype signatures. The *STRESS* genes identified patients with a significantly shorter RFS when compared with other genetic signatures of PDAC. Considering that Moffitt, Collisson, and subsequent molecular subtypes^52–54^ reflect cell types in their immediate biological state, we propose that understanding how cancer cells adapt to dynamic changes in the tumor microenvironment is key for predicting future tumor behavior and developing more efficacious treatment strategies. Furthermore, a cell’s ability to adapt to its environment is governed by its capacity to activate specific gene expression programs, with not all cells possessing the necessary prerequisites to be able to do so.^52–59^ Accordingly, the *STRESS* gene signature has the potential to complement previously identified PDAC gene signatures because it describes which tumors are likely to adapt to stress, inflammation, and other selective pressures independent of their molecular subtype. Indeed, we empirically demonstrate that *STRESS* inducibility is independent of known molecular subtypes in human PDAC cell lines. Similarly, the *STRESS* signature did not correlate with PDAC subtypes in patients, suggesting that PDAC tumors, regardless of their molecular subtype, contain individual cells capable of exhibiting a *STRESS* response. While this observation appears to conflict with a correlation between *ITGB3* expression and basal/Q.M. subtypes, we propose two explanations for this discrepancy. First, as reported by Werba et al.,^18^ each patients’ tumor has heterogeneous populations of classical and basal cells, so a gain of *STRESS* genes may not be exclusive to either subtype but instead reflect adaptations of individual cells to the evolving landscape of the tumor microenvironment. Second, given that STAT3 signaling is not exclusive to tumor cells, the *STRESS* signature may reflect contributions from multiple cell types in the tumor microenvironment.

Finally, like the Collisson and Moffitt molecular subtypes, the *STRESS* and *STRESS-Up* signatures are more strongly associated with poor survival for pancreatic cancer than for other epithelial cancers. Future efforts could replicate our process to define signatures to reflect the role of STAT3 in the adaptation to stress including targeted therapeutics or chemotherapies, in other types of cancers, or in chronic inflammatory conditions that are linked to cancer but for which cells do not typically harbor oncogenic mutations. It would also be interesting to identify the drivers of the epigenetic states that allow or deny STAT3 recruitment to gene enhancer “families” and to explore how to interfere with this process selectively and effectively without interrupting STAT3’s broader functions.

### Limitations of the study

This study primarily models stress and inflammation using hypoxia and IL-6 family cytokines, which serve as simplified proxies for more complex physiological processes. Nevertheless, previous studies, including our own, demonstrate that diverse stressors, such as nutrient deprivation and oxidative stress from drug treatment, can induce the expression of integrin β3, suggesting broader relevance. Our focus on early transcriptional responses emphasizes mechanisms occurring well before typical clinical diagnosis, raising questions about how these findings translate to established disease. While integrin β3 and *STRESS* signature genes are transiently expressed during periods of stress and inflammation and decline once tumors are established, their fleeting expression may belie their functional importance. Indeed, we found that these genes are critical for tumor initiation at the primary site; however, while preliminary evidence suggests that integrin β3 and *STRESS* signature genes may also be important for metastatic seeding, further studies are required. Although integrin β3 plays a prominent role, the contributions of other *STRESS* genes to tumor initiation, stress adaptation, and progression remain unclear and will be the subject of a future study. Importantly, while we used genetic perturbations to interrogate the STAT3/β3 axis, we did not explicitly test whether pharmacologic inhibition of STAT3 can block tumor initiation or progression *in vivo*. Moreover, although we showed that chemotherapy induces *STRESS* genes in human tumors, we could not demonstrate whether targeting this pathway could sensitize tumors to standard-of-care treatment. Thus, while our findings highlight a novel opportunity to stratify PDAC subpopulations for STAT3-targeted therapies, additional work is needed to validate this approach.

## RESOURCE AVAILABILITY

### Lead contact

Requests for further information and resources and reagents should be directed to and will be fulfilled by the lead contact, Dr. David Cheresh (dcheresh@health.ucsd.edu).

### Materials availability

All unique reagents generated during this study are available from the lead contact with a completed materials transfer agreement.

### Data and code availability

- ChIP-seq data generated for this paper have been deposited in the NCBI BioProject database under accession number BioProject: PRJNA1170024 and are publicly available as of the date of publication.
- All data reported in this paper will be shared by the lead contact upon request.
- This paper does not report original code.
- Any additional information required to reanalyze the data reported in this paper is available from the lead contact upon request.

## STAR★METHODS

Detailed methods are provided in the online version of this paper and include the following:

### EXPERIMENTAL MODEL AND STUDY PARTICIPANT DETAILS

#### Cell culture

Human cancer cell lines were obtained from commercial vendors along with proof of cell line authentication. Upon receipt, each cell line was expanded, cryopreserved as low-passage stocks, and tested for mycoplasma using a MycoScope PCR Mycoplasma Detection Kit (Genlantis, MY01050). Cell lines were used for 30 passages or less. FG, CFPAC, PANC1, and MIAPACA2 cell lines were cultured in DMEM supplemented with 10% FBS and 2 mM L-Glutamine [2 mM]. The remaining cell lines were cultured in RPMI supplemented with 10% FBS and 2 mM L-Glutamine [2 mM].

#### Mouse study approval

All experiments involving mice were conducted under protocol S05018, approved by the UC San Diego Institutional Animal Care and Use Committee. All experiments were performed in accordance with the NIH Guide for the Care and Use of Laboratory Animals. All animals were housed under standard conditions, i.e., given unrestricted access to food and water, housed in standard cages, and rooms regulated to control temperature and light cycles. Cell lines used for *in vivo* experiments confirmed negative for a panel of human pathogens. Age-matched littermates were assigned randomly to experimental groups.

#### Subcutaneous xenograft model

Human pancreatic cancer cells were injected subcutaneously to mice in 100 μL sterile HBSS in 6- to 8-week-old female immune-compromised nu/nu mice (Charles River Labs). Mice were examined twice weekly for palpable tumors. Time to tumor initiation was determined by the emergence of a tumor larger than 100 mm^3^ in volume, using Volume = 0.5 × Length × Width^2^.

#### i-KPC and iKPC-β3KO mice

*β3*^*flox/flox*^ mice^69^ were obtained from Dr. Katherine Weilbaecher at Washington University St. Louis. i-KPC (*Kras*^*LSL-G12D*^*/Tp53*^*flox/flox*^*/Pdx1-Cre*^*ER*^; Strain #032429) were acquired from Jackson Labs, maintained on a B6 background for several generations, and crossed to generate i-KPC-B3KO mice (*Kras*^*LSL*−G12D^/*Tp53*^*flox*/flox/^*Itgb3*^*flox*/flox^/*Pdx1-Cre*^*ER*^). Adult β3 WT and KO siblings (age-matched littermates of either sex) were treated I.P. at post-natal days 42, 44, and 46 with 225 mg/kg Tamoxifen (Sigma; T5648-1G) in corn oil to induce recombination. For survival/end-stage experiments, mice were sacrificed when they became moribund, and pancreas tissue and tumors were collected and either Zinc-formalin fixed for histology and/or flash frozen for RNA analysis. To assess early-stage tumor-initiating events, mice were sacrificed and the pancreas collected for histology 60 days following Tamoxifen treatment. No sex-differences were observed.

### METHOD DETAILS

#### Histology

Zinc-formalin fixed tissues were deparaffinized and rehydrated followed by antigen retrieval in high pH buffer at 95°C. Tissues were stained using the ImmPRESS Excel Staining Kit (VectorLabs, MP-7601-50) and developed using DAB or the ImmPACT VIP Substrate Kit (VectorLabs, SK-4605). Primary antibodies for integrin β3 (Cell Signaling, 13166S, 1:200) and KRT19 (Invitrogen, MA5-35221, 1:200) were incubated overnight at 4°C. Sections were also stained for H&E (VectorLabs, H-3502) and Alcian blue (VectorLabs, H-3501) to assess pathology. The slides were imaged using an Olympus VS200 Slide Scanner at The UCSD School of Medicine Light Microscopy Facility (funded by grant NINDS P30NS047101). Whole tissue quantification was performed by a blinded observer using QuPath software for Bioimage Analysis.^69^

#### Immunofluorescence staining

Fresh frozen pancreatic tissues from KTC and KTC-Stat3KO mice were sectioned onto glass slides and aerosol fixed for 15 min with 10% normal buffered formalin. Tissues were subsequently dried on a slide warmer for 20 min to remove moisture and ensure adhesion to the slides. High pH antigen retrieval was performed at 95°C for 25 min to unmask epitopes, followed by incubation with Mouse-on-Mouse Blocking Reagent (VectorLabs, MKB-2213-1), 2.5% normal horse serum, and primary antibodies overnight against CPA1 (R&D, AF2765, 1:400), KRT19 (Genetex, GTX27755, 1:150), and integrin β3 (Cell Signaling, 13166S, 1:200). Proteins were visualized using species-specific fluorescence-conjugated secondary antibodies. Images were obtained using an Olympus VS200 slide scanner, and whole tissue quantification was performed by a blinded observer using QuPath software.

#### Immunoblotting

Immunoblotting was performed as previously described. Briefly, cells were washed twice with HBSS before lysing with either 1X RIPA buffer containing protease and phosphatase inhibitors, 2X sample buffer containing 1X reducing agent, or for conditioned supernatant, 4X sample buffer containing 1X reducing agent (BioRad #1610737 and #1610747). A BCA assay (Thermo, 23227) was performed, and the lysates were normalized. Sample buffer (NuPAGE LDS Sample Buffer 4X, Sigma, Catalog#NP0007) and reducing agent (NuPAGE Sample Reducing Agent, Sigma, NP0009) were added to the cell lysates. All samples were heated at 95°C for 5 min 10 μg of protein or 30 μL of each sample containing Laemmli buffer was loaded onto an SDS-PAGE gel. Blocking was performed in 5% BSA in TBS-T, and probing was performed in 5% BSA in TBST buffer.

#### Effect of cytokines on β3 and αvβ3 expression levels

Human recombinant cytokines were purchased from PeproTech: OSM (300–10H; 0.2 ng/ml [WB]; 10 ng/ml [FACS]), LIF (300–05; 10 ng/ml [WB]; 20 ng/ml [FACS]), IL-11 (200–11; 10 ng/ml), CNTF (450–13; 10 ng/ml), NNT-1 (450–18; 10 ng/ml), CT1 (300–32; 10 ng/ml), IL-27 (200–38; 10 ng/ml), IL-31 (200–31; 10 ng/ml), TGF-β1 (100–21; 10 ng/ml); Bio-techne: Il-6-sIL-6R (8954-SR-025; 5 ng/ml [WB]; 20 ng/ml [FACS]); and Proteintech: IL-6 (HZ-1019–100UG; 10 ng/ml). Cells were cultured under standard conditions with or without the addition of inflammatory cytokines for 72 h, then either lysed to evaluate β3 protein expression using immunoblot, or else dissociated into a single cell suspension, stained for αvβ3 surface expression with LM609 (1 ng/ml), and assessed using flow cytometry. The JAK inhibitors, ruxolitinib and baricitinib, and the histone deacetylase inhibitor Vorinostat were reconstituted in DMSO and administered to cells at indicated concentrations and 0.1% DMSO. JAK inhibitors and Vorinostat were administered simultaneously with inflammatory cytokines for 72 h. Total cell lysates were then prepared for immunoblot analysis.

#### Effect of hypoxic stress on β3 and αvβ3 expression levels

Cells were cultured under normal atmospheric oxygen levels (normoxia) or hypoxic conditions using a Coy Oxygen Control In-Vitro Glove Box (hypoxia chamber), which maintains oxygen levels at 1% to model a hypoxic environment. Cells were cultured in normoxia or hypoxia for 72 h before being lysed and prepared for immunoblot analysis. To investigate the effects hypoxia mediated effects on αvβ3 surface expression, cells were cultured in normoxia or hypoxia for 96 h. Media was changed after 48 h. JAK inhibitors were added to cells just prior to moving to cells into the hypoxia chamber.

#### Effect of STAT3 knockdown on hypoxia-stimulated β3 expression

STAT3 expression was silenced using small interfering RNA (siRNA) which was resuspended in 1X siRNA buffer (Horizon cat#: B-002000-UB-100). Target cells plated 24 h earlier were transfected with siRNA targeting STAT3 or a non-targeting control to a final concentration of 50 nM with the lipofectamine 2000 transfection reagent (ThermoFisher cat#: 11668019) per the manufacturer’s instructions. Cells were incubated for 48 h before media was changed and cells were relocated to the hypoxia chamber. Cells were then incubated in hypoxia for 72 h before being removed, lysed, and prepared for immunoblot analysis.

#### qPCR analysis

To assess RNA expression in tumors from mice, 100 mg flash frozen tissues were homogenized in Buffer RLT (Qiagen), and RNA was purified using the RNeasy mini kit (Qiagen cat# 74106). For experiments using human cell lines, adherent (2D) cells were washed twice with 1× HBSS and scraped off in the presence of Buffer RLT (Qiagen). Complementary DNA was synthesized by using High-Capacity cDNA Reverse Transcription Kit (Thermo Fisher Scientific #4368814), and RT–PCR was performed on a Bio-Rad thermocycler with SYBR Green (Bio-Rad #1725124). Expression of each target gene is normalized to a stable housekeeping gene and quantified using the 2−ΔΔCT method. Validated primer sequences from Harvard Primer Bank were synthesized by Integrated DNA Technologies and are listed in Tables S1–S3.

#### Establishment of CRISPR KO cell lines

CRISPR knockout (KO) cell lines were established by transiently transfecting FG cells with CAS9-2A-GFP and an individual and gene specific CRISPR guide strand plasmid using the lipofectamine 2000 transfection agent following the manufactures guidelines.

Successfully transfected can be visualized 48 h later as green fluorescent protein (GFP) positive cells under a fluorescent microscope. 72 h after transfection, GFP+ cells were FACS sorted into a 96 well plate (1 cell/well). Single cell clones were allowed to expand. Positive clones for target KO were selected by immunoblot analysis for either STAT3 or β3 knockout. Genetic edits were confirmed by Sanger sequencing (data not shown). Experimental KO lines represent pools of a minimum of six validated clones.

#### Establishment of cell lines expressing ectopic β3

Lentivirus was generated in Hek293T packaging cell line after transfecting the Integrin β3 Lentiviral transfer plasmid (GeneCopoeia) and packaging plasmids for PAX2 and VSVG with lipofectamine 2000. Functional virus secreted from HEK293T cells was collected and filtered through 0.45 μm cellulose acetate membrane. FG target cells were then transduced with the filtered virus +8[μg/mL] polybrene overnight. Positively transduced cells were selected for with puromycin [2 μg/mL]. Ectopic expression of β3 was confirmed by immunoblot and flow cytometry analysis.

#### Flow cytometry

For flow cytometry, cell pellets were washed with PBS, blocked with 1% BSA in PBS for 30 min at room temperature, and stained with or without indicated primary antibodies with fluorescently labeled secondary antibodies. Cells were incubated with live/dead fixable blue dead cell stain kit (Invitrogen, L23105). Flow cytometry was performed on a BD Fortessa X-20 (BD) analyzer, and the data were analyzed using FlowJo (Treestar) software.

#### ChIP-seq

STAT3 ChIP-seqs were carried out as described previously^82^ double-crosslinking cells with 2 mM disuccinimidyl glutarate/PBS and 1% formaldehyde final, and performing sonication and IP using RIPA buffer.

#### ChIP-seq analysis

ChIP-seq was analyzed using two independent pipelines by independent researchers. First, ChIP FASTQ files were aligned to Hg38 reference genome using bowtie2 and the resulting SAM alignment files were converted to tag directories using the makeTagDirectory command of the HOMER suite.^12^ To visualize ChIP-seq data, bedGraph files were created for each tag directory using HOMER’s makeUSCfile command. STAT3 binding regions were then identified with HOMER findPeaks command. This command produced a peaks file for each sample in both the control and experimental groups. Differential peaks between hypoxia vs. normoxia groups and OSM treated vs. non-treated cells were then identified by comparing individual sample peak files. The differential peaks were identified and annotated using the HOMER getDifferentialPeaks. Differential peaks were annotated using HOMER annotatePeaks and GeneHancer database. Differential peaks were then ranked by the peak score generated by HOMER getDifferentialPeaks analysis. Due to the excessive number of peaks identified, we selected the top 200 ranked peaks identified form hypoxia and OSM experiments. Peaks present in both datasets were identified using the Bedtools intersect feature.

To ensure the findings are robust and unbiased, ChIP FASTQ files were aligned to Hg38 reference genome using bowtie2 and the resulting SAM alignment files were converted to BAM files. Mitochondrial reads were removed using samtools. To visualize read pile-ups with IGV, bigwig files were generated using the bamCoverage command. Peaks were found with MACS3 and quantified with featureCounts. Differential expression of counts was conducted with DEseq2. Peaks were annotated with HOMER annotatePeaks.pl and GeneHancer. Differential peaks were defined as adjusted *p* < 0.05.

#### ChIP-PCR

STAT3 and Histone H3-K27 acetylation ChIP-PCRs were carried after double-crosslinking cells with 2 mM disuccinimidyl glutarate/PBS and 1% formaldehyde. Chromatin sonication was performed with Covaris E220 focused ultrasonicator as described previously^82^ and IPs were performed using SimpleChIP^®^ Chromatin IP Buffers (Cell Signaling Cat #14231) per the manufacturer recommendations.

#### β3 inducible signature identification

Expression data for 5 PDAC cancer cell lines empirically determined to be inducible and 5 cell lines determined to be non-inducible was downloaded from the Cancer Cell Line Encyclopedia (CCLE) database via the DepMap web portal. We accessed the raw counts for these 10 cell lines and performed a differential expression analysis using the DESeq2 bioinformatics package in R. Differentially expressed genes (DEGs) were identified as genes with Log2 fold changes >1 or < −1 and an adjusted *p* value (FDR) < 0.05. The DEGs were visualized as a volcano plot and as heatmap generated using the EnhancedVolcano (https://bioconductor.org/packages/release/bioc/html/EnhancedVolcano.html) and pheatmap (https://cran.r-project.org/web/packages/pheatmap/index.html) packages in R. The DESeq2 normalized expression counts were extracted and rlog transformed. DEGs with standard deviations >2 in either the inducible and non-inducible groups were filtered out in order to generate a robust signature that includes only differentially expressed genes common to all cell lines in each group. The 93 remaining upregulated DEGs were used to define the β3 inducible signature.

#### ATAC-seq analysis

In order to determine if differences in gene expression were regulated by chromatin accessibility, publicly available ATAC-seq FASTQ files representing empirically tested “Inducible” and “Non-inducible” cell lines were downloaded from the NCBI GEO/SRA Repositories. FASTQ files were aligned to Hg38 using bowtie2. Multimapping sequences and mitochondrial reads were removed using samtools. Duplicate and unmapped reads and reads with low quality were removed with picard MarkDuplicates. To visualize read pile-ups with IGV, bigwig files were generated using the bamCoverage command. Peaks were found with MACS2. Differential expression of accessible peaks was conducted with DEseq2. Peaks were annotated with HOMER annotatePeaks.pl and GeneHancer.

#### *STRESS* signature identification

To find differentially accessible chromatin sites in common to the STAT3 binding sites identified in previous CHIP-seq experiments, bed files for ChIP- and ATAC-seq files were filtered using the Bedtools Intersect feature. Regions identified as open chromatin and STAT3 binding (STAT-open regions) were identified and annotated with GeneHancer. 54 genes associated with these identified regions were cataloged. The computeMatrix (https://deeptools.readthedocs.io/en/develop/content/tools/computeMatrix.html) R package was used to illustrate that all the observed STAT-open regions were located at enhancer regions. Gene expression data from the TCGA PAAD patient cohort was downloaded. Expression data for the 54 cataloged genes was extracted for each patient and Pearson correlations were performed for every possible gene-pair using the ggcorrplot (https://cran.r-project.org/web/packages/ggcorrplot/) R package. The calculated *p* values were extracted and adjusted for multiple testing using the Holm method. After filtering out gene pairs with *p* values < 0.05, we calculated and ranked how often individual genes were represented in significantly correlated gene pairs. We selected the most correlated genes as genes ranked in the half of our rankings. Correlation plots were generated with the ggplot2 (https://ggplot2.tidyverse.org/) R package. The correlation analysis enabled us to filter the 54 gene list down to 18. Of these 18 genes, 10 were positively correlated and 8 were negatively correlated. The 10 positively correlated genes represented STAT3 inducible genes and the remaining 8 represented STAT3 repressed genes. The 10 inducible genes were selected as the *STRESS* signature.

#### Survival analysis

In order to evaluate the correlation between individual gene expression or average expression of gene sets with human patient survival, we utilized the online survival analysis tool KM-plotter.^32^ The Pan-cancer mRNA RNAseq Pancreatic ductal adenocarcinoma cohort of patients (*n* = 177) derived from GEO, EGA, and TCGA repositories was used to assess Overall and Relapse-free survival utilizing the following parameters: Use multiple genes feature; Use mean expression of selected genes; Invert feature for downregulated genes; Auto select best cutoff; Compute median survival; Censore at threshold. Hazard ratios and *p* values are reported.

#### Single-cell RNA sequencing analysis of human PDACs

Single-cell RNA sequencing of human PDACs, including both untreated and treated samples, were analyzed. Data were retrieved from the Gene Expression Omnibus Series GSE205013,^18^ which includes 27 pre- and post-chemotherapy samples from primary and liver metastatic sites. For this analysis, *n* = 12 primary tumors were selected, including *n* = 6 untreated and *n* = 6 treated samples.

Unprocessed.mtx files were downloaded and pooled based on treatment status into our analysis pipeline built using scanpy^77^ and scvi-tools.^78^ Cells expressing fewer than 200 genes and genes detected in fewer than 10 cells were excluded. Doublets were identified and removed using the SOLO model from the scVI package. Mitochondrial (MT) and ribosomal gene expression metrics were assessed: cells with MT and ribosomal gene counts exceeding more than 15% of a cell’s total gene pool were removed. Cells with abnormally high gene counts, above a 98th percentile threshold also were excluded.

Following sample processing, ribosomal genes were removed to reduce noise and enhance the detection of biologically relevant expression patterns. Batch correction and normalization were applied to the pooled sample sets using an scVI-trained model. The library size-normalized gene values were then used to calculate nearest-neighbors and the Leiden algorithm (resolution = 0.1) was applied for clustering, followed by Uniform Manifold Approximation Projection (UMAP) visualization. Initial automated cluster annotation was performed using the CellTypist package with the ‘Immune_All_Low.pkl’ model.^79^ For the *n* = 12 Leiden clusters, the rank_genes_groups function in Scanpy was used to identify the top marker genes for each cluster. After assigning predicted labels, the cluster annotations were finalized through manual verification using known marker genes from PanglaoDB.^80^ As an additional filtering step, only cells labeled as ‘Epithelial cells’ by CellTypist’s ‘majority_voting’ prediction from the malignant UMAP clusters were retained for downstream analysis.

Copy number variation (CNV) analysis was performed on the epithelial cell subsets using inferCNV,^81^ with the immune cell population designated as the normal reference to establish baseline gene expression. A sliding window of 250 genes was applied to calculate CNV scores on a per-cell basis. The lowest CNV scoring epithelial cell cluster, representing normal-adjacent tissue, was removed for downstream analysis.

To explore the effects of *ITGB3* expression, epithelial cells were stratified based on gene expression levels for the entire malignant sample pool. The top 10% (*ITGB3*-High) and bottom 10% (*ITGB3*-Low) of cells, representing the highest and lowest expression extremes, were selected for analysis. This resulted in 2,270 cells for each group (high and low expression) across both treatment conditions. Gene set scoring using the scanpy score_genes function was used to assess differences in STAT3 genes identified from ChIP sequencing, and basal, classical, and quasi-mesenchymal (QM) PDAC subtypes. Distribution of signature scores for each *ITGB3* population were visualized as a violin plot, and a Mann-Whitney U statistic was calculated for each to validate statistical significance.

### QUANTIFICATION AND STATISTICAL ANALYSIS

All quantification and statistical analyses were performed either with GraphPad Prism or R, except for human patient survival analyses which were performed with KM plotter. The statistical tests used, the value of *n*, what *n* represents, the graph metrics, and the significance values are indicated in the figure legends and/or on the figures directly. Statistical significance was set to *p* < 0.05 unless otherwise stated.

## Supplementary Material

1

SUPPLEMENTAL INFORMATION

Supplemental information can be found online at https://doi.org/10.1016/j.celrep.2025.116010.

## Figures and Tables

**KEY RESOURCES TABLE:** 

REAGENT or RESOURCE	SOURCE	IDENTIFIER
Antibodies		
Integrin β3	Cell Signaling	Cat#: 13166S; RRID: AB_2798136
STAT3	Cell Signaling	Cat#: 4904; RRID: AB_331269
STAT3-pY705	Cell Signaling	Cat#: 9131; RRID: AB_331586
STAT3-pY705 (used for ChIP)	Cell Signaling	Cat#: 9145; RRID: AB_2491009
Acetyl-Histone H3 (Lys27) (D5E4) XP^®^	Cell Signaling	Cat#: 8173; RRID: AB_10949503
Acetyl-Histone H3 (Lys9) (C5B11)	Cell Signaling	Cat#: 9649; RRID: AB_823528
β-actin	Santa Cruz	Cat#: sc-47778; RRID: AB_626632
Integrin αvβ3 (clone LM609)	Millipore Sigma	Cat#: MAB1976; RRID: AB_2296419
KRT19	Genetex	Cat#: GTX27755; RRID: AB_369536
CPA1	R&D Systems	Cat#: AF2765; RRID: AB_2085841
Bacterial and virus strains		
DH5α	Fisher	Cat#: 18258012
Biological samples		
Fresh frozen pancreatic tissues from KTC and KTC-Stat3KO mice	Dr. Valerie Weaver^23^	UCSF
Chemicals, peptides, and recombinant proteins		
Ruxolitinib	Selleckchem	S1378
Baricitinib	Selleckchem	S2851
Vorinostat	Selleckchem	S1047
OSM	PeproTech	300-10H
LIF	PeproTech	300-05
IL-6	Proteintech	HZ-1019-100UG
IL-6-sIL-6R	Bio-Techne	8954-SR-025
IL-11	PeproTech	200-11
CNTF	PeproTech	450-13
NNT-1	PeproTech	450-18
CT1	PeproTech	300-32
IL-27	PeproTech	200-38
IL-31	PeproTech	200-31
TGF-β	PeproTech	100-21
Tamoxifen	Sigma	T5648-1G
Hematoxylin and Eosin stain (H + E)	VectorLabs	H-3502
Alcian blue stain	VectorLabs	H-3501
Critical commercial assays		
MycoScope PCR Mycoplasma Detection Kit	Genlantis	MY01050
Mouse on Mouse Blocking Reagent	VectorLabs	MKB-2213-1
ImmPRESS Excel Staining Kit	VectorLabs	MP-7601-50
ImmPACT VIP Substrate Kit	VectorLabs	SK-4605
Hypoxyprobe-Green Kit	Hypoxyprobe	HP6-100Kit
RNeasy Mini Kit	Qiagen	74106
High-Capacity cDNA Reverse Transcription Kit	Thermo Fisher Scientific	4368814
iTaq^™^ Universal SYBR^®^ Green Supermix	Bio-Rad	1725124
ChIP-Grade Protein G Magnetic Beads	Cell Signaling	9006
SimpleChIP^®^ Chromatin IP Buffers	Cell Signaling	14231
ATAC-seq data (CAPAN2)	Tu et al.^60^	SRR12357401 SRR12357402 BioProject: PRJNA649804
ATAC-seq data (MIAPACA2)	Alfarano et al.^61^	GSM5058791 GSM5058792 BioProject: PRJNA698630
ATAC-seq data (ASPC1)	Lee et al.^62^	GSM5799840 GSM5799841 BioProject: PRJNA796043
ATAC-seq data (SUIT2)	Tsai et al.^63^	GSM4298978 GSM4298979 BioProject: PRJNA605101
ATAC-seq data (HCC827)	Criscione et al.^64^	SRR17490272 SRR17490271 BioProject: PRJNA795501
ATAC-seq data (H1650)	Pierce et al.^65^	SRR13774045 SRR13774046 BioProject: PRJNA704434
scRNA-seq data of human PDAC	Werber et al.^18^	GSE20501384
pSTAT3-Y705 ChIP-seq data	This paper	BioProject: PRJNA1170024
Experimental models: Cell lines		
KP4	RIKEN BRC	RCB1005
KP2	JCRB Cell Bank	JCRB0181
CFPAC1	ATCC	CRL-1918; RRID: CVCL_1119
PANC1	ATCC	CRL-1469; RRID: CVCL_0480
COLO-357/FG (“FG”)	Dr. Shama Kajiji and Vito Quaranta	The Scripps Research Institute
SUIT2	JCRB Cell Bank	JCRB1094; RRID: CVCL_3172
HPAFII	ATCC	CRL-1997; RRID: CVCL_0313
CAPAN1	ATCC	HTB-79; RRID: CVCL_0237
DANG	DSMZ	ACC 249; RRID: CVCL_0243
BxPC3	ATCC	CRL-1687; RRID: CVCL_0186
CAPAN2	ATCC	HTB-80; RRID: CVCL_0026
ASPC1	ATCC	CRL-1682; RRID: CVCL_0152
MIAPACA2	ATCC	CRL-1420; RRID: CVCL_0428
SW1990	ATCC	CRL-2172; RRID: CVCL_1723
Experimental models: Organisms/strains		
Immune-compromised nu/nu mice (Crl:NU-*Foxn1^nu^*)	Charles River Labs	088
i-KPC (KrasLSL-G12D/Tp53flox/flox/Pdx1-CreER)	Jackson Labs	032429
*β3-integrin-floxed mice* (*β3^flox/flox^*)	Dr. Katherine Weilbaecher^66^	Washington University (St. Louis)
i-KPC-B3KO mice (i-KPC crossed with *β3^flox/flox^* mice)	This paper	N/A
Oligonucleotides		
*STAT3 CRISPR targeting sequence-1* AAGGCGTGATTCTTCCCAC	Custom oligo gBlocks were made by IDT	N/A
*STAT3 CRISPR targeting sequence-1* GAACAGATGCTCACTGCGC	Custom oligo gBlocks were made by IDT	N/A
STAT3 targeting siRNA (ON-TARGETplus siRNA)	Horizon Discovery	L-003544-00-0005
Non-targeting siRNA control (ON-TARGETplus siRNA)	Horizon Discovery	D-001810-10-05
upstream (GH17J047239) Forward primer for quantitative PCR of pSTAT Y705 binding GAGACATGCTTGCTAATCATACTTCC	Custom oligo were made by IDT	N/A
upstream (GH17J047239) Reverse primer for quantitative PCR of pSTAT Y705 binding CACCAGAAAAACCTGGGCATTGGGCC	Custom oligo were made by IDT	N/A
downstream (GH17J047305) Forward primer for quantitative PCR of pSTAT Y705 binding CTGACATTTGTGGGAAGCAGAGC	Custom oligo were made by IDT	N/A
downstream (GH17J047305) Reverse primer for quantitative PCR of pSTAT Y705 binding CCAGTCAACAACTAAAACAGAGG	Custom oligo were made by IDT	N/A
upstream (GH17J047239) Forward primer for quantitative of PCR of H3K27acetylation binding CCACAGTGCCCCAAATATCCATTATT	Custom oligo were made by IDT	N/A
upstream (GH17J047239) Reverse primer for quantitative PCR of H3K27 acetylation binding AGAGGCCAAACACTCAATAGTCAAGCC	Custom oligo were made by IDT	N/A
downstream (GH17J047305) Forward primer for quantitative PCR of H3K27 acetylation binding GAAAGGTTAAGTAATTTTCCAAAGATC	Custom oligo were made by IDT	N/A
downstream (GH17J047305) Reverse primer for quantitative PCR of H3K27 acetylation binding GTCTGCGCTCCTGTGGATGTTACAGCC	Custom oligo were made by IDT	N/A
Recombinant DNA		
Human *ITGB3*	GeneCopoeia	EX-E2219-Lv105
pReceiver-Lv105 empty vector control	GeneCopoeia	EX-Lv105
CRISPR for *ITGB3* guide strand plasmids *Plasmids were obtained from Hiromi* gs1: ACCTCGCGTGGTACAGATGT gs2: CCCAACATCTGTACCACGCGG	Plasmids were provided by Dr. Hiromi Wettersten^67^	UC San Diego
CRISPR for *STAT3* guide strand plasmids *Plasmids were designed with protocol outlined here*^68^	Plasmids were generated in house	UC San Diego
CMV-CAS9-2A-GFP Plasmid	Millipore-Sigma	CAS9GFPP-1EA
Software and algorithms		
QuPath software	Bankhead et al.^69^	https://qupath.github.io
bowtie2 software	Langmead and Salzberg^70^	https://bowtie-bio.sourceforge.net/bowtie2/index.shtml
Samtools software	Danecek et al.^71^	https://www.htslib.org/
picard MarkDuplicates	picard	https://broadinstitute.github.io/picard/
deepTools bamCoverage	Ramírez et al.^72^	https://deeptools.readthedocs.io/en/develop/content/tools/bamCoverage.html
MACS2	Gaspar^73^	https://github.com/jsh58/MACS
DESeq2	Love et al.^74^	https://bioconductor.org/packages/release/bioc/html/DESeq2.html
HOMER	Heinz et al.^12^	http://homer.ucsd.edu/homer/ngs/annotation.html
Bedtools Intersect	Bedtools	https://bedtools.readthedocs.io/en/latest/content/tools/intersect.html
deepTools computeMatrix	Ramírez et al.^72^	https://deeptools.readthedocs.io/en/develop/content/tools/computeMatrix.html
ggcorrplot		https://cran.r-project.org/web/packages/ggcorrplot/
ggplot2	Wickham^75^	https://ggplot2.tidyverse.org/
SingScore	Foroutan et al.^76^	https://bioconductor.org/packages/release/bioc/html/singscore.html
scanpy	Wolf et al.^77^	https://github.com/broadinstitute/infercnv?tab=readme-ov-file
scvi-tools	Gayoso et al.^78^	https://scvi-tools.org/
CellTypist	Xu et al.^79^	https://www.celltypist.org/
PanglaoDB	Franzen et al.^80^	http://panglaodb.se/
inferCNV	Tickle et al.^81^	https://github.com/broadinstitute/infercnv
FlowJo v10	BD Biosciences	https://www.flowjo.com/
GraphPad Prism v9.2.0	Dotmatics	https://www.graphpad.com/
R version 4.3.3	The Comprehensive R Archive Network	https://cran.r-project.org/

## Highlights

- Integrin β3 is a STAT3-regulated stress/inflammatory response gene
- The STAT3/β3 axis accelerates tumor initiation and promotes the basal PDAC subtype
- Accessibility of enhancer regions to STAT3 controls the expression of integrin β3
- STAT3-receptive enhancers identify patient tumors adapting to stress and inflammation

## References

[1] Aboelella NS, Brandle C, Kim T, Ding ZC, and Zhou G (2021). Oxidative Stress in the Tumor Microenvironment and Its Relevance to Cancer Immunotherapy. Cancers (Basel) 13, 986. 10.3390/cancers13050986. 33673398 PMC7956301

[2] Gómez-Valenzuela F, Escobar E, Pérez-Tomás R, and Montecinos VP (2021). The Inflammatory Profile of the Tumor Microenvironment, Orchestrated by Cyclooxygenase-2, Promotes Epithelial-Mesenchymal Transition. Front. Oncol. 11, 686792. 10.3389/fonc.2021.686792. 34178680 PMC8222670

[3] Li K, Deng Z, Lei C, Ding X, Li J, and Wang C (2024). The Role of Oxidative Stress in Tumorigenesis and Progression. Cells 13, 441. 10.3390/cells13050441. 38474405 PMC10931308

[4] Nishida A, and Andoh A (2025). The Role of Inflammation in Cancer: Mechanisms of Tumor Initiation, Progression, and Metastasis. Cells 14, 488. 10.3390/cells14070488. 40214442 PMC11987742

[5] Tolomeo M, and Cascio A (2021). The Multifaced Role of STAT3 in Cancer and Its Implication for Anticancer Therapy. Int. J. Mol. Sci. 22, 603. 10.3390/ijms22020603. 33435349 PMC7826746

[6] Al-Hetty HRAK, Abdulameer SJ, Alkubaisy SA, Zaid SA, Jalil AT, and Jasim IK (2023). STAT3 signaling in pancreatic ductal adenocarcinoma: a candidate therapeutic target. Pathol. Res. Pract. 245, 154425. 10.1016/j.prp.2023.154425. 37019018

[7] Corcoran RB, Contino G, Deshpande V, Tzatsos A, Conrad C, Benes CH, Levy DE, Settleman J, Engelman JA, and Bardeesy N (2011). STAT3 plays a critical role in KRAS-induced pancreatic tumorigenesis. Cancer Res. 71, 5020–5029. 10.1158/0008-5472.Can-11-0908. 21586612 PMC3693754

[8] Zhao Y, Qin C, Zhao B, Wang Y, Li Z, Li T, Yang X, and Wang W (2023). Pancreatic cancer stemness: dynamic status in malignant progression. J. Exp. Clin. Cancer Res. 42, 122. 10.1186/s13046-023-02693-2. 37173787 PMC10182699

[9] Seguin L, Kato S, Franovic A, Camargo MF, Lesperance J, Elliott KC, Yebra M, Mielgo A, Lowy AM, Husain H, (2014). An integrin β_3_-KRAS-RalB complex drives tumour stemness and resistance to EGFR inhibition. Nat. Cell Biol. 16, 457–468. 10.1038/ncb2953. 24747441 PMC4105198

[10] Desgrosellier JS, Barnes LA, Shields DJ, Huang M, Lau SK, Prévost N, Tarin D, Shattil SJ, and Cheresh DA (2009). An integrin alpha (v)beta(3)-c-Src oncogenic unit promotes anchorage-independence and tumor progression. Nat. Med. 15, 1163–1169. 10.1038/nm.2009. 19734908 PMC2759406

[11] Cosset É, Ilmjärv S, Dutoit V, Elliott K, von Schalscha T, Camargo MF, Reiss A, Moroishi T, Seguin L, Gomez G, (2017). Glut3 Addiction Is a Druggable Vulnerability for a Molecularly Defined Subpopulation of Glioblastoma. Cancer Cell 32, 856–868.e5. 10.1016/j.ccell.2017.10.016. 29198914 PMC5730343

[12] Heinz S, Benner C, Spann N, Bertolino E, Lin YC, Laslo P, Cheng JX, Murre C, Singh H, and Glass CK (2010). Simple combinations of lineage-determining transcription factors prime cis-regulatory elements required for macrophage and B cell identities. Mol. Cell 38, 576–589. 10.1016/j.molcel.2010.05.004. 20513432 PMC2898526

[13] Fishilevich S, Nudel R, Rappaport N, Hadar R, Plaschkes I, Iny Stein T, Rosen N, Kohn A, Twik M, Safran M, (2017). GeneHancer: genome-wide integration of enhancers and target genes in GeneCards. Database 2017, bax028. 10.1093/database/bax028. 28605766 PMC5467550

[14] Desgrosellier JS, Lesperance J, Seguin L, Gozo M, Kato S, Franovic A, Yebra M, Shattil SJ, and Cheresh DA (2014). Integrin αvβ3 drives slug activation and stemness in the pregnant and neoplastic mammary gland. Dev. Cell 30, 295–308. 10.1016/j.devcel.2014.06.005. 25117682 PMC4147869

[15] Nam A, Jain S, Wu C, Campos A, Shepard RM, Yu Z, Reddy JP, Von Schalscha T, Weis SM, Onaitis M, (2024). Integrin αvβ3 Upregulation in Response to Nutrient Stress Promotes Lung Cancer Cell Metabolic Plasticity. Cancer Res. 84, 1630–1642. 10.1158/0008-5472.Can-23-2700. 38588407 PMC11096068

[16] Seguin L, Camargo MF, Wettersten HI, Kato S, Desgrosellier JS, von Schalscha T, Elliott KC, Cosset E, Lesperance J, Weis SM, and Cheresh DA (2017). Galectin-3, a Druggable Vulnerability for KRAS-Addicted Cancers. Cancer Discov. 7, 1464–1479. 10.1158/2159-8290.Cd-17-0539. 28893801 PMC5718959

[17] Zhang T, Zhang Z, Dong Q, Xiong J, and Zhu B (2020). Histone H3K27 acetylation is dispensable for enhancer activity in mouse embryonic stem cells. Genome Biol. 21, 45. 10.1186/s13059-020-01957-w. 32085783 PMC7035716

[18] Werba G, Weissinger D, Kawaler EA, Zhao E, Kalfakakou D, Dhara S, Wang L, Lim HB, Oh G, Jing X, (2023). Single-cell RNA sequencing reveals the effects of chemotherapy on human pancreatic adenocarcinoma and its tumor microenvironment. Nat. Commun. 14, 797. 10.1038/s41467-023-36296-4. 36781852 PMC9925748

[19] Chiang FF, Huang SC, Yu PT, Chao TH, and Huang YC (2023). Oxidative Stress Induced by Chemotherapy: Evaluation of Glutathione and Its Related Antioxidant Enzyme Dynamics in Patients with Colorectal Cancer. Nutrients 15, 5104. 10.3390/nu15245104. 38140363 PMC10745799

[20] Reyes-Castellanos G, Abdel Hadi N, Gallardo-Arriaga S, Masoud R, Garcia J, Lac S, El Kaoutari A, Gicquel T, Planque M, Fendt SM, (2023). Combining the antianginal drug perhexiline with chemotherapy induces complete pancreatic cancer regression *in vivo*. iScience 26, 106899. 10.1016/j.isci.2023.106899. 37305702 PMC10250830

[21] Moffitt RA, Marayati R, Flate EL, Volmar KE, Loeza SGH, Hoadley KA, Rashid NU, Williams LA, Eaton SC, Chung AH, (2015). Virtual microdissection identifies distinct tumor- and stroma-specific subtypes of pancreatic ductal adenocarcinoma. Nat. Genet. 47, 1168–1178. 10.1038/ng.3398. 26343385 PMC4912058

[22] Collisson EA, Sadanandam A, Olson P, Gibb WJ, Truitt M, Gu S, Cooc J, Weinkle J, Kim GE, Jakkula L, (2011). Subtypes of pancreatic ductal adenocarcinoma and their differing responses to therapy. Nat. Med. 17, 500–503. 10.1038/nm.2344. 21460848 PMC3755490

[23] Laklai H, Miroshnikova YA, Pickup MW, Collisson EA, Kim GE, Barrett AS, Hill RC, Lakins JN, Schlaepfer DD, Mouw JK, (2016). Genotype tunes pancreatic ductal adenocarcinoma tissue tension to induce matricellular fibrosis and tumor progression. Nat. Med. 22, 497–505. 10.1038/nm.4082. 27089513 PMC4860133

[24] Maddipati R, and Stanger BZ (2015). Pancreatic Cancer Metastases Harbor Evidence of Polyclonality. Cancer Discov. 5, 1086–1097. 10.1158/2159-8290.Cd-15-0120. 26209539 PMC4657730

[25] Mori S, Kodaira M, Ito A, Okazaki M, Kawaguchi N, Hamada Y, Takada Y, and Matsuura N (2015). Enhanced Expression of Integrin αvβ3 Induced by TGF-β Is Required for the Enhancing Effect of Fibroblast Growth Factor 1 (FGF1) in TGF-β-Induced Epithelial-Mesenchymal Transition (EMT) in Mammary Epithelial Cells. PLoS One 10, e0137486. 10.1371/journal.pone.0137486. 26334633 PMC4559424

[26] Wingelhofer B, Neubauer HA, Valent P, Han X, Constantinescu SN, Gunning PT, Müller M, and Moriggl R (2018). Implications of STAT3 and STAT5 signaling on gene regulation and chromatin remodeling in hematopoietic cancer. Leukemia 32, 1713–1726. 10.1038/s41375-018-0117-x. 29728695 PMC6087715

[27] Swaroop S, Batabyal A, and Bhattacharjee A (2021). HAT/HDAC: The epigenetic regulators of inflammatory gene expression (Review). Int. J. Epigen. 1, 5. 10.3892/ije.2021.5.

[28] Mansisidor AR, and Risca VI (2022). Chromatin accessibility: methods, mechanisms, and biological insights. Nucleus 13, 236–276. 10.1080/19491034.2022.2143106. 36404679 PMC9683059

[29] Tsherniak A, Vazquez F, Montgomery PG, Weir BA, Kryukov G, Cowley GS, Gill S, Harrington WF, Pantel S, Krill-Burger JM, (2017). Defining a Cancer Dependency Map. Cell 170, 564–576. e16. 10.1016/j.cell.2017.06.010. 28753430 PMC5667678

[30] Bruns CJ, Harbison MT, Kuniyasu H, Eue I, and Fidler IJ (1999). In vivo selection and characterization of metastatic variants from human pancreatic adenocarcinoma by using orthotopic implantation in nude mice. Neoplasia 1, 50–62. 10.1038/sj.neo.7900005. 10935470 PMC1764837

[31] Jin X, Demere Z, Nair K, Ali A, Ferraro GB, Natoli T, Deik A, Petronio L, Tang AA, Zhu C, (2020). A metastasis map of human cancer cell lines. Nature 588, 331–336. 10.1038/s41586-020-2969-2. 33299191 PMC8439149

[32] Győrffy B (2024). Integrated analysis of public datasets for the discovery and validation of survival-associated genes in solid tumors. Innovation 5, 100625. 10.1016/j.xinn.2024.100625. 38706955 PMC11066458

[33] Lin W, Noel P, Borazanci EH, Lee J, Amini A, Han IW, Heo JS, Jameson GS, Fraser C, Steinbach M, (2020). Single-cell transcriptome analysis of tumor and stromal compartments of pancreatic ductal adenocarcinoma primary tumors and metastatic lesions. Genome Med. 12, 80. 10.1186/s13073-020-00776-9. 32988401 PMC7523332

[34] Bailey P, Chang DK, Nones K, Johns AL, Patch AM, Gingras MC, Miller DK, Christ AN, Bruxner TJC, Quinn MC, (2016). Genomic analyses identify molecular subtypes of pancreatic cancer. Nature 531, 47–52. 10.1038/nature16965. 26909576

[35] Yu H, Kortylewski M, and Pardoll D (2007). Crosstalk between cancer and immune cells: role of STAT3 in the tumour microenvironment. Nat. Rev. Immunol. 7, 41–51. 10.1038/nri1995. 17186030

[36] Rébé C, and Ghiringhelli F (2019). STAT3, a Master Regulator of Anti-Tumor Immune Response. Cancers (Basel) 11, 1280. 10.3390/cancers11091280. 31480382 PMC6770459

[37] D’Amico S, Shi J, Martin BL, Crawford HC, Petrenko O, and Reich NC (2018). STAT3 is a master regulator of epithelial identity and KRAS-driven tumorigenesis. Genes Dev. 32, 1175–1187. 10.1101/gad.311852.118. 30135074 PMC6120712

[38] Hamel Z, Sanchez S, Standing D, and Anant S (2024). Role of STAT3 in pancreatic cancer. Explor Target Antitumor Ther. 5, 20–34. 10.37349/etat.2024.00202. 38464736 PMC10918236

[39] Hu L, Zang MD, Wang HX, Zhang BG, Wang ZQ, Fan ZY, Wu H, Li JF, Su LP, Yan M, (2018). G9A promotes gastric cancer metastasis by upregulating ITGB3 in a SET domain-independent manner. Cell Death Dis. 9, 278. 10.1038/s41419-018-0322-6. 29449539 PMC5833452

[40] Sun F, Wang J, Sun Q, Li F, Gao H, Xu L, Zhang J, Sun X, Tian Y, Zhao Q, (2019). Interleukin-8 promotes integrin β3 upregulation and cell invasion through PI3K/Akt pathway in hepatocellular carcinoma. J. Exp. Clin. Cancer Res. 38, 449. 10.1186/s13046-019-1455-x. 31684995 PMC6829822

[41] Seguin L, Desgrosellier JS, Weis SM, and Cheresh DA (2015). Integrins and cancer: regulators of cancer stemness, metastasis, and drug resistance. Trends Cell Biol. 25, 234–240. 10.1016/j.tcb.2014.12.006. 25572304 PMC4380531

[42] Liu Z, Han L, Dong Y, Tan Y, Li Y, Zhao M, Xie H, Ju H, Wang H, Zhao Y, (2016). EGFRvIII/integrin β3 interaction in hypoxic and vitronectinenriching microenvironment promote GBM progression and metastasis. Oncotarget 7, 4680–4694. 10.18632/oncotarget.6730. 26717039 PMC4826235

[43] Xu YH, Li ZL, and Qiu SF (2018). IFN-γ Induces Gastric Cancer Cell Proliferation and Metastasis Through Upregulation of Integrin β3-Mediated NF-κB Signaling. Transl. Oncol. 11, 182–192. 10.1016/j.tranon.2017.11.008. 29306706 PMC5755748

[44] Wen S, Hou Y, Fu L, Xi L, Yang D, Zhao M, Qin Y, Sun K, Teng Y, and Liu M (2019). Cancer-associated fibroblast (CAF)-derived IL32 promotes breast cancer cell invasion and metastasis via integrin β3-p38 MAPK signalling. Cancer Lett. 442, 320–332. 10.1016/j.canlet.2018.10.015. 30391782

[45] Hieken TJ, Farolan M, Ronan SG, Shilkaitis A, Wild L, and Das Gupta TK (1996). Beta3 integrin expression in melanoma predicts subsequent metastasis. J. Surg. Res. 63, 169–173. 10.1006/jsre.1996.0242. 8661192

[46] Bauer TM, Patel MR, Forero-Torres A, George TJ Jr., Assad A, Du Y, and Hurwitz H (2018). A Phase Ib study of ruxolitinib + gemcitabine ± nab-paclitaxel in patients with advanced solid tumors. OncoTargets Ther. 11, 2399–2407. 10.2147/ott.S157331. PMC5935192 29750040

[47] Hurwitz H, Van Cutsem E, Bendell J, Hidalgo M, Li CP, Salvo MG, Macarulla T, Sahai V, Sama A, Greeno E, (2018). Ruxolitinib + capecitabine in advanced/metastatic pancreatic cancer after disease progression/intolerance to first-line therapy: JANUS 1 and 2 randomized phase III studies. Invest. New Drugs 36, 683–695. 10.1007/s10637-018-0580-2. 29508247 PMC6752723

[48] Lee M, Hirpara JL, Eu JQ, Sethi G, Wang L, Goh BC, and Wong AL (2019). Targeting STAT3 and oxidative phosphorylation in oncogene-addicted tumors. Redox Biol. 25, 101073. 10.1016/j.redox.2018.101073. 30594485 PMC6859582

[49] Datta J, Dai X, Bianchi A, De Castro Silva I, Mehra S, Garrido VT, Lamichhane P, Singh SP, Zhou Z, Dosch AR, (2022). Combined MEK and STAT3 Inhibition Uncovers Stromal Plasticity by Enriching for Cancer-Associated Fibroblasts With Mesenchymal Stem Cell-Like Features to Overcome Immunotherapy Resistance in Pancreatic Cancer. Gastroenterology 163, 1593–1612. 10.1053/j.gastro.2022.07.076. 35948109 PMC10257389

[50] Stupp R, Hegi ME, Gorlia T, Erridge SC, Perry J, Hong YK, Aldape KD, Lhermitte B, Pietsch T, Grujicic D, (2014). Cilengitide combined with standard treatment for patients with newly diagnosed glioblastoma with methylated MGMT promoter (CENTRIC EORTC 26071–22072 study): a multicentre, randomised, open-label, phase 3 trial. Lancet Oncol. 15, 1100–1108. 10.1016/s1470-2045(14)70379-1. 25163906

[51] Alday-Parejo B, Stupp R, and Rüegg C (2019). Are Integrins Still Practicable Targets for Anti-Cancer Therapy? Cancers (Basel) 11, 978. 10.3390/cancers11070978. 31336983 PMC6678560

[52] Lomberk G, Blum Y, Nicolle R, Nair A, Gaonkar KS, Marisa L, Mathison A, Sun Z, Yan H, Elarouci N, (2018). Distinct epigenetic landscapes underlie the pathobiology of pancreatic cancer subtypes. Nat. Commun. 9, 1978. 10.1038/s41467-018-04383-6. 29773832 PMC5958058

[53] Somerville TDD, Xu Y, Miyabayashi K, Tiriac H, Cleary CR, Maia-Silva D, Milazzo JP, Tuveson DA, and Vakoc CR (2018). TP63-Mediated Enhancer Reprogramming Drives the Squamous Subtype of Pancreatic Ductal Adenocarcinoma. Cell Rep. 25, 1741–1755.e7. 10.1016/j.celrep.2018.10.051. 30428345 PMC6296757

[54] Li Y, He Y, Peng J, Su Z, Li Z, Zhang B, Ma J, Zhuo M, Zou D, Liu X, (2021). Mutant Kras co-opts a proto-oncogenic enhancer network in inflammation-induced metaplastic progenitor cells to initiate pancreatic cancer. Nat. Cancer 2, 49–65. 10.1038/s43018-020-00134-z. 35121887

[55] Brunton H, Caligiuri G, Cunningham R, Upstill-Goddard R, Bailey UM, Garner IM, Nourse C, Dreyer S, Jones M, Moran-Jones K, (2020). HNF4A and GATA6 Loss Reveals Therapeutically Actionable Subtypes in Pancreatic Cancer. Cell Rep. 31, 107625. 10.1016/j.celrep.2020.107625. 32402285 PMC9511995

[56] Todoric J, and Karin M (2019). The Fire within: Cell-Autonomous Mechanisms in Inflammation-Driven Cancer. Cancer Cell 35, 714–720. 10.1016/j.ccell.2019.04.001. 31085174

[57] Buenrostro JD, Wu B, Litzenburger UM, Ruff D, Gonzales ML, Snyder MP, Chang HY, and Greenleaf WJ (2015). Single-cell chromatin accessibility reveals principles of regulatory variation. Nature 523, 486–490. 10.1038/nature14590. 26083756 PMC4685948

[58] Vierbuchen T, Ling E, Cowley CJ, Couch CH, Wang X, Harmin DA, Roberts CWM, and Greenberg ME (2017). AP-1 Transcription Factors and the BAF Complex Mediate Signal-Dependent Enhancer Selection. Mol. Cell 68, 1067–1082.e12. 10.1016/j.molcel.2017.11.026. 29272704 PMC5744881

[59] Alonso-Curbelo D, Ho YJ, Burdziak C, Maag JLV, Morris JP 4th, Chandwani R, Chen HA, Tsanov KM, Barriga FM, Luan W, (2021). A gene-environment-induced epigenetic program initiates tumorigenesis. Nature 590, 642–648. 10.1038/s41586-020-03147-x. 33536616 PMC8482641

[60] Tu M, Klein L, Espinet E, Georgomanolis T, Wegwitz F, Li X, Urbach L, Danieli-Mackay A, Küffer S, Bojarczuk K, (2021). TNF-α-producing macrophages determine subtype identity and prognosis via AP1 enhancer reprogramming in pancreatic cancer. Nat. Cancer 2, 1185–1203. 10.1038/s43018-021-00258-w. 35122059

[61] Alfarano G, Audano M, Di Chiaro P, Balestrieri C, Milan M, Polletti S, Spaggiari P, Zerbi A, Diaferia GR, Mitro N, and Natoli G (2023). Interferon regulatory factor 1 (IRF1) controls the metabolic programmes of low-grade pancreatic cancer cells. Gut 72, 109–128. 10.1136/gutjnl-2021-325811. 35568393

[62] Lee MS, Dennis C, Naqvi I, Dailey L, Lorzadeh A, Ye G, Zaytouni T, Adler A, Hitchcock DS, Lin L, (2023). Ornithine aminotransferase supports polyamine synthesis in pancreatic cancer. Nature 616, 339–347. 10.1038/s41586-023-05891-2. 36991126 PMC10929664

[63] Tsai PY, Lee MS, Jadhav U, Naqvi I, Madha S, Adler A, Mistry M, Naumenko S, Lewis CA, Hitchcock DS, (2021). Adaptation of pancreatic cancer cells to nutrient deprivation is reversible and requires glutamine synthetase stabilization by mTORC1. Proc. Natl. Acad. Sci. USA 118, e2003014118. 10.1073/pnas.2003014118. 33653947 PMC7958225

[64] Criscione SW, Martin MJ, Oien DB, Gorthi A, Miragaia RJ, Zhang J, Chen H, Karl DL, Mendler K, Markovets A, (2022). The landscape of therapeutic vulnerabilities in EGFR inhibitor osimertinib drug tolerant persister cells. npj Precis. Oncol. 6, 95. 10.1038/s41698-022-00337-w. PMC9794691 36575215

[65] Pierce SE, Granja JM, Corces MR, Brady JJ, Tsai MK, Pierce AB, Tang R, Chu P, Feldser DM, Chang HY, (2021). LKB1 inactivation modulates chromatin accessibility to drive metastatic progression. Nat. Cell Biol. 23, 915–924. 10.1038/s41556-021-00728-4. 34341533 PMC8355205

[66] Morgan EA, Schneider JG, Baroni TE, Uluçkan O, Heller E, Hurchla MA, Deng H, Floyd D, Berdy A, Prior JL, (2010). Dissection of platelet and myeloid cell defects by conditional targeting of the beta3-integrin subunit. FASEB J. 24, 1117–1127. 10.1096/fj.09-138420. 19933310 PMC2845430

[67] Wettersten HI, Weis SM, Pathria P, Von Schalscha T, Minami T, Varner JA, and Cheresh DA (2019). Arming Tumor-Associated Macrophages to Reverse Epithelial Cancer Progression. Cancer Res. 79, 5048–5059. 10.1158/0008-5472.Can-19-1246. 31416839 PMC6774806

[68] Yang L, Yang JL, Byrne S, Pan J, and Church GM (2014). CRISPR/Cas9-Directed Genome Editing of Cultured Cells. Curr. Protoc. Mol. Biol. 107, 31.1.1–31.1.17. 10.1002/0471142727.mb3101s107. 24984853

[69] Bankhead P, Loughrey MB, Fernández JA, Dombrowski Y, McArt DG, Dunne PD, McQuaid S, Gray RT, Murray LJ, Coleman HG, (2017). QuPath: Open source software for digital pathology image analysis. Sci. Rep. 7, 16878. 10.1038/s41598-017-17204-5. 29203879 PMC5715110

[70] Langmead B, and Salzberg SL (2012). Fast gapped-read alignment with Bowtie 2. Nat. Methods 9, 357–359. 10.1038/nmeth.1923. 22388286 PMC3322381

[71] Danecek P, Bonfield JK, Liddle J, Marshall J, Ohan V, Pollard MO, Whitwham A, Keane T, McCarthy SA, Davies RM, and Li H (2021). Twelve years of SAMtools and BCFtools. GigaScience 10, giab008. 10.1093/gigascience/giab008. 33590861 PMC7931819

[72] Ramiŕez F, Dündar F, Diehl S, Grüning BA, and Manke T (2014). deepTools: a flexible platform for exploring deep-sequencing data. Nucleic Acids Res. 42, W187–W191. 10.1093/nar/gku365. 24799436 PMC4086134

[73] Gaspar JM (2018). Improved peak-calling with MACS2. Preprint at: bioRxiv. 10.1101/496521

[74] Love MI, Huber W, and Anders S (2014). Moderated estimation of fold change and dispersion for RNA-seq data with DESeq2. Genome Biol. 15, 550. 10.1186/s13059-014-0550-8. 25516281 PMC4302049

[75] Wickham H (2016). ggplot2: Elegant Graphics for Data Analysis (Springer-Verlag).

[76] Foroutan M, Bhuva DD, Lyu R, Horan K, Cursons J, and Davis MJ (2018). Single sample scoring of molecular phenotypes. BMC Bioinf. 19, 404. 10.1186/s12859-018-2435-4. PMC6219008 30400809

[77] Wolf FA, Angerer P, and Theis FJ (2018). SCANPY: large-scale single-cell gene expression data analysis. Genome Biol. 19, 15. 10.1186/s13059-017-1382-0. 29409532 PMC5802054

[78] Gayoso A, Lopez R, Xing G, Boyeau P, Valiollah Pour Amiri V, Hong J, Wu K, Jayasuriya M, Mehlman E, Langevin M, (2022). A Python library for probabilistic analysis of single-cell omics data. Nat. Biotechnol. 40, 163–166. 10.1038/s41587-021-01206-w. 35132262

[79] Xu C, Prete M, Webb S, Jardine L, Stewart BJ, Hoo R, He P, Meyer KB, and Teichmann SA (2023). Automatic cell-type harmonization and integration across Human Cell Atlas datasets. Cell 186, 5876–5891.e20. 10.1016/j.cell.2023.11.026. 38134877

[80] Franzén O, Gan LM, and Björkegren JLM (2019). PanglaoDB: a web server for exploration of mouse and human single-cell RNA sequencing data. Database 2019, baz046. 10.1093/database/baz046. 30951143 PMC6450036

[81] Tickle T, Tirosh I, Georgescu C, Brown M, and Haas B (2019). inferCNV of the Trinity CTAT Project (Klarman Cell Observatory, Broad Institute of MIT and Harvard). https://github.com/broadinstitute/inferCNV.

[82] Texari L, Spann NJ, Troutman TD, Sakai M, Seidman JS, and Heinz S (2021). An optimized protocol for rapid, sensitive and robust on-bead ChIP-seq from primary cells. STAR Protoc. 2, 100358. 10.1016/j.xpro.2021.100358. 33718886 PMC7921621

