# Supplementary material for: A STAT3/integrin axis accelerates pancreatic cancer initiation and progression
Source: Cell Rep. Author manuscript; Available in PMC 2025 Sep 18. (PMC12445075; doi:10.1016/j.celrep.2025.116010)
Supplement: 1 [file NIHMS2107243-supplement-1.pdf]

Cell Reports, Volume *44*

Supplemental information

A STAT3/integrin axis accelerates pancreatic cancer initiation and progression

Alejandro D. Campos, Ryan M. Shepard, Zachary Ortega, Ingrid Heumann, Anna E. Wilke, Arin Nam, Carson Cable, Kourosh Kouhmareh, Richard Klemke, Nicole M. Mattson, Trey Ideker, Camila De Arruda Saldanha, Sven Heinz, Valerie Weaver, Tami Von Schalscha, Hiromi I. Wettersten, Sara M. Weis, and David A. Cheresh

Figure S1. related to Figure 1

A **JAK/STAT3 signaling is high in TCGA PAAD Patient Cohort**

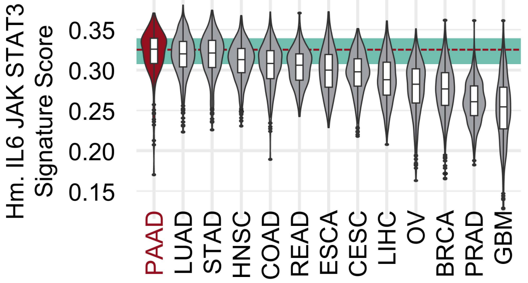

B **STAT3 binds to both DNA promoter regions and enhancer regions**

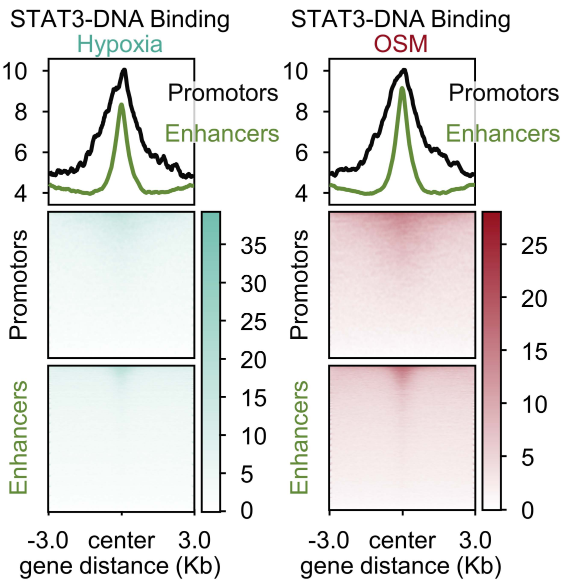

C **Stress response transcription factors have enriched binding motifs in STAT3 peaks**

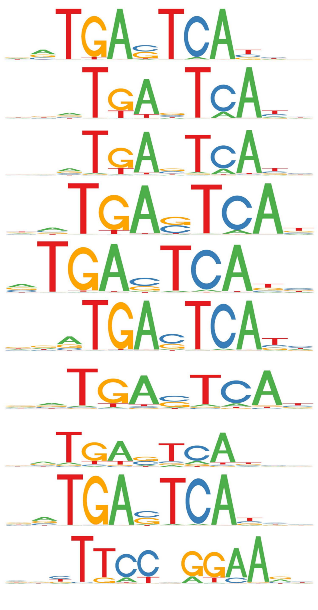
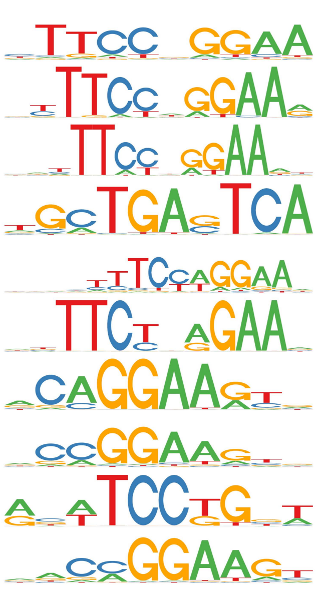

Rank	p-value	Motif	Transcription Factor
1	1e-293		ATF3
2	1e-285		FOS
3	1e-282		FRA1
4	1e-278		BATF
5	1e-267		AP1
6	1e-260		FRA2
7	1e-256		JUNB
8	1e-203		FOSL2
9	1e-169		JUN-AP1
10	1e-126		STAT3+IL21
11	1e-121		STAT3
12	1e-90		STAT4
13	1e-78		STAT1
14	1e-59		BACH2
15	1e-53		BCL6
16	1e-44		STAT5
17	1e-32		ERG
18	1e-27		ETV4
19	1e-27		SPDEF
20	1e-26		ETV1

D ***ITGB3* mRNA expression stimulated by treatment with inflammatory cytokines and cellular stress**

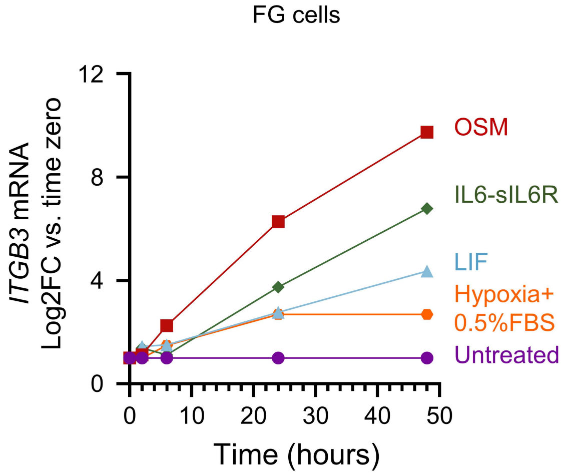

E **The combination of hypoxia and IL6-family cytokines have an additive effect on the induction of integrin β3**

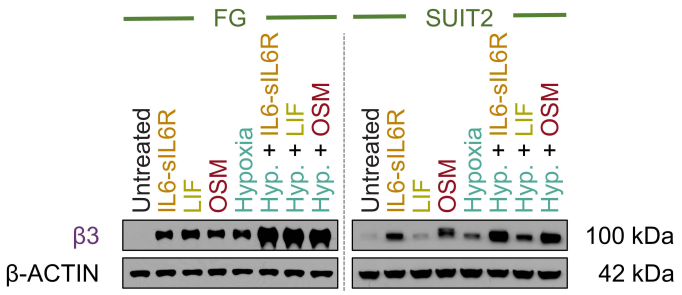

Figure S1. related to Figure 1

A. Violin plots show the distribution of patient Hallmark IL6 JAK STAT3 Signaling gene set scores for RNA sequencing of patient tumors in The Cancer Genome Atlas (TCGA).

B. Genomic distribution of STAT3 binding between promoter regions and enhancer regions

C. Motif enrichment in STAT3 peaks

D. Graph shows log2-fold change for *ITGB3* mRNA expression for FG cells treated with OSM, IL6-sIL6R, LIF, or subjected to hypoxia (1% O_2) combined with 0.5% fetal bovine serum (FBS), relative to untreated/control FG cells.

E. FG and SUIT2 cells were treated with vehicle control, IL6-sIL6R, LIF, or OSM in both normoxia and hypoxia conditions. Cell lysates were immunoblotted to detect expression of integrin β3.

Figure S2. related to Figure 3

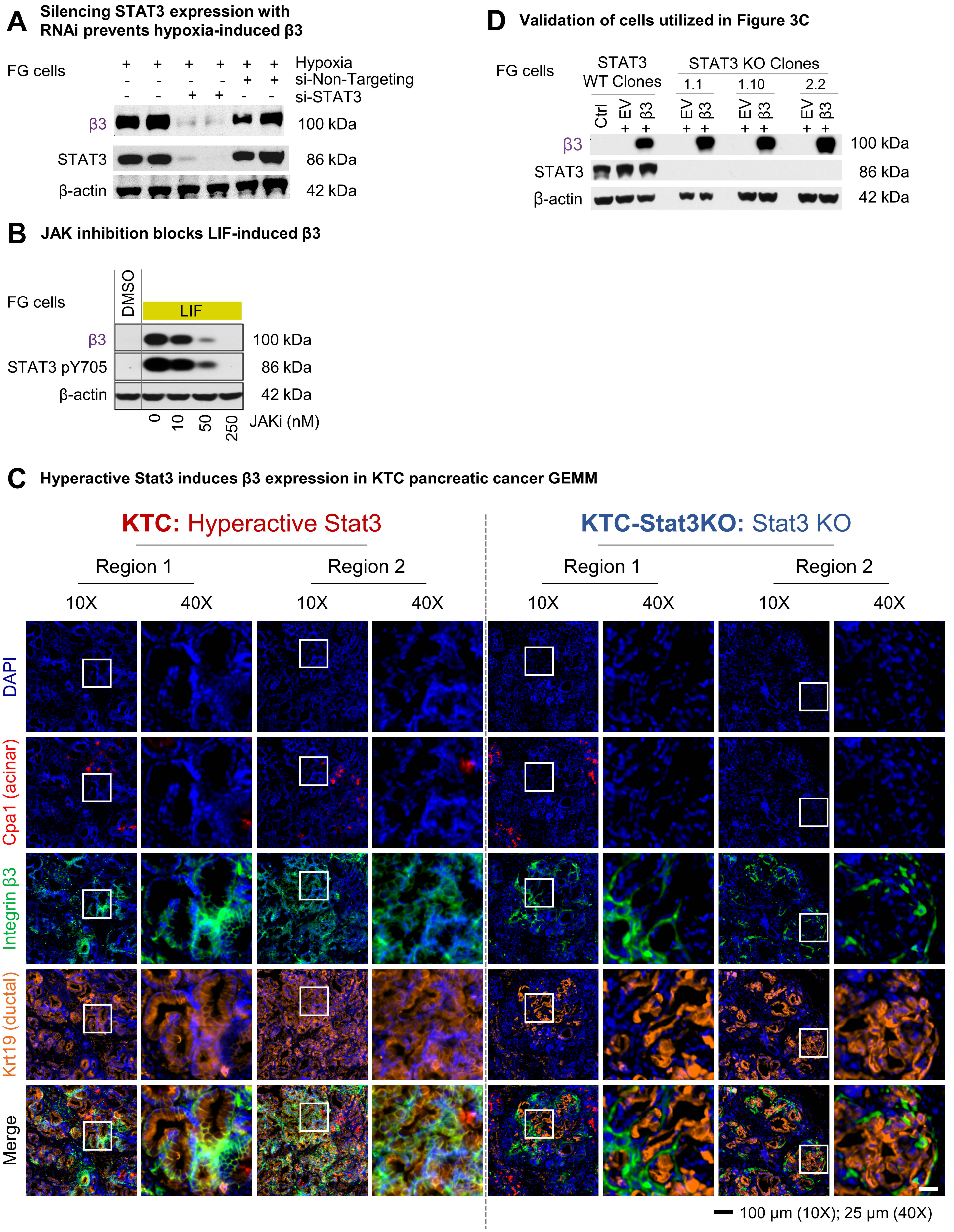

Figure S2. related to Figure 3

A. FG cells were challenged with hypoxia (1% O2) for 72 hours, and β3 expression evaluated by immunoblot for cells with siRNA-mediated silencing of STAT3 vs. a non-silencing scramble control.

B. Immunoblot shows expression of β3 and STAT3 pY705 for FG cells stimulated with 10 ng/mL LIF for 72 hours in the presence of 10-250 nM of the JAK inhibitor ruxolitinib.

C. Images of KTC vs. KTC-Stat3KO tumors shown in Figure 3B are included here at two magnifications (10X and 40X) to demonstrate 4-color staining for the nuclear marker DAPI (blue), the acinar marker CPA1 (red), integrin β3 (green), the ductal marker KRT19 (orange), and the merge of these 4 channels. Scale bars represent 100 μm (10X); 25 μm (40X).

D. To validate the cell lines utilized in Figure 3C, immunoblots show STAT3 and β3 expression levels for FG CRISPR WT control cells vs. *STAT3* CRISPR KO clones with ectopic expression of empty vector (+EV) or ITGB3 (+β3).

Figure S3. related to Figure 4

A Validation of cells used in Figure 4A

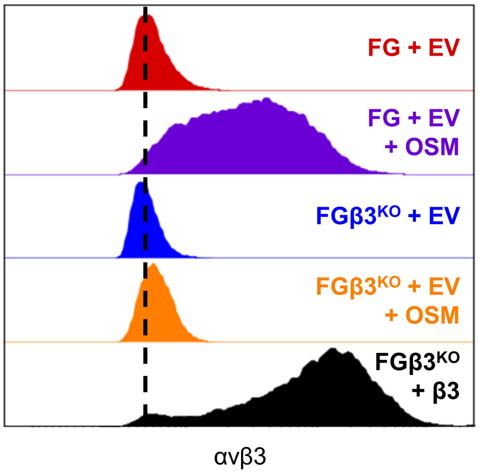

B Early and late stage histology of i-KPC mouse model

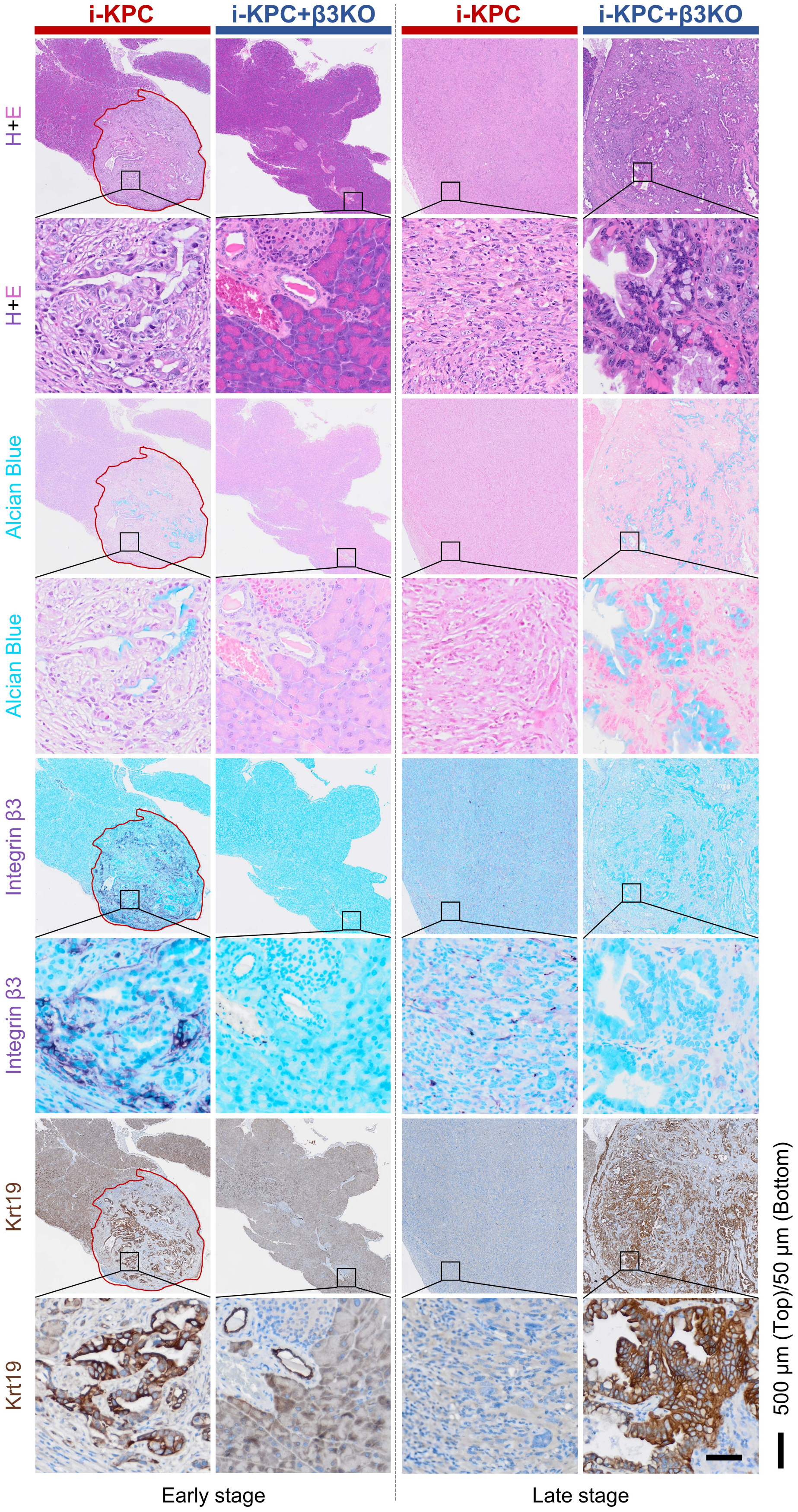

Figure S3. related to Figure 4

A. To validate cells utilized in Figure 4A, flow cytometry was performed to quantify integrin αvβ3 cell surface expression for FG cells and $FG\beta3^{KO}$ cells with or without OSM treatment or ectopic β3 expression.

B. Representative images of early and late-stage histology of i-KPC and i-KPC-β3KO mice. Serial sections were stained with hematoxylin and eosin (H+E), alcian blue, integrin β3 (purple) and KRT19 (brown). Scale bars represent 500 μm (Top)/50 μm (Bottom/magnification)

Figure S4. related to Figure 6

A **Lack of response to OSM predicts response to expanded panel of cytokines**

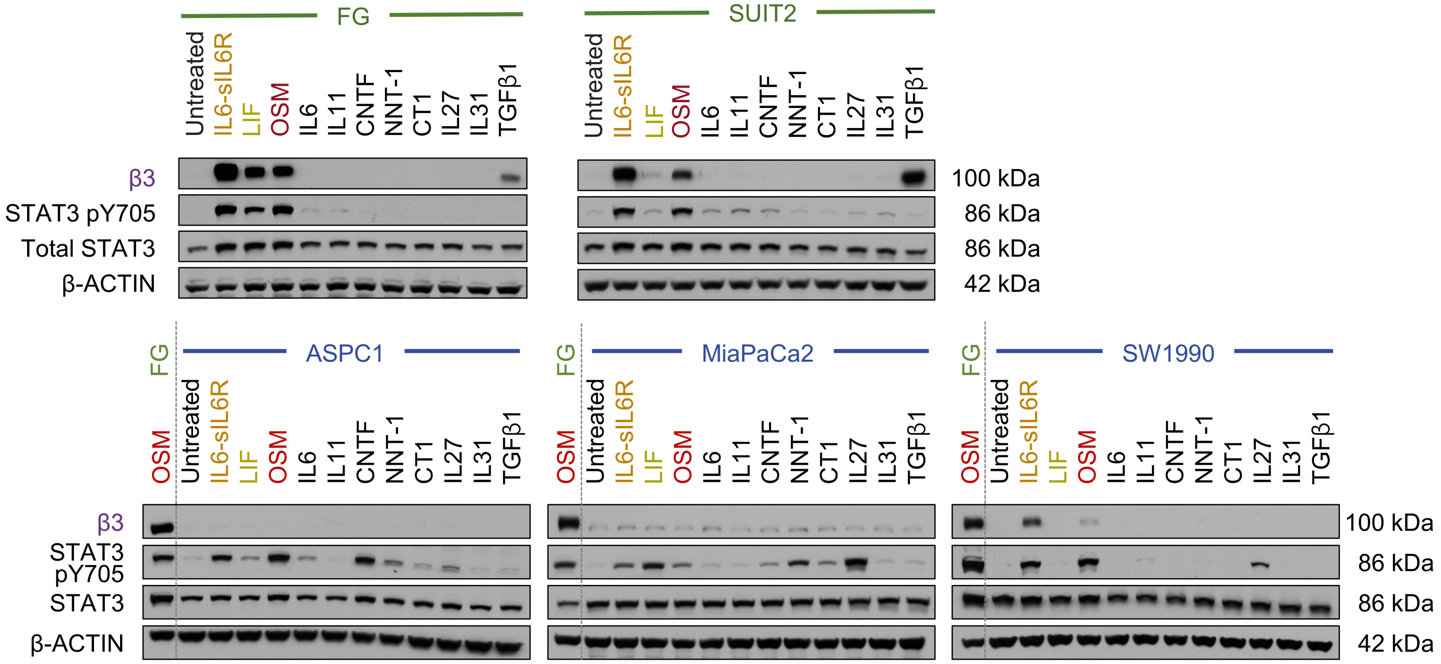

B **Chromatin at upstream and downstream enhancers for *ITGB3* is closed in "non-inducible" cells**

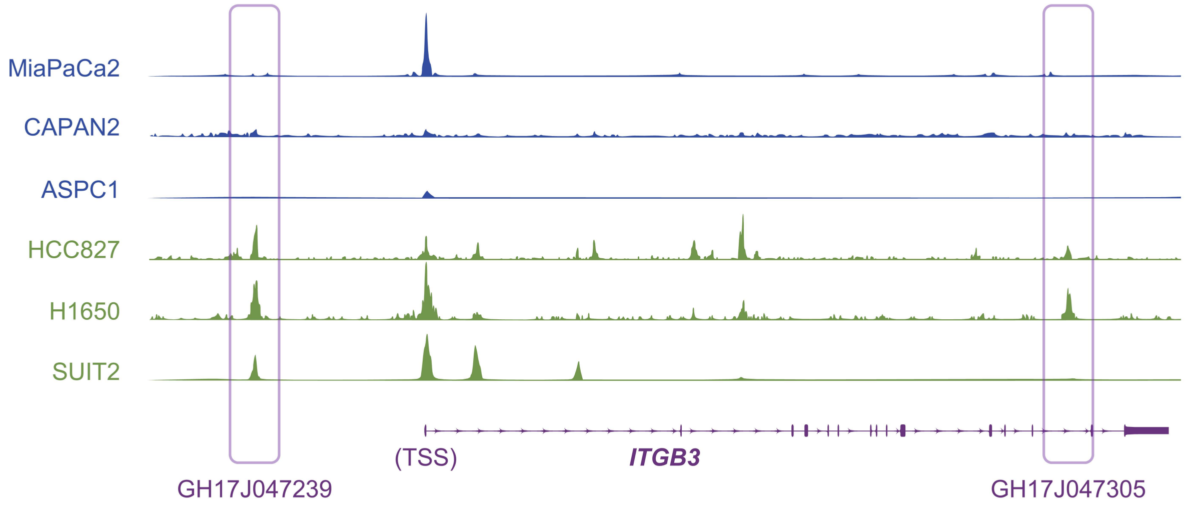

C **Confirmation of β3 inducibility by OSM for H1650 and HCC827 cell lines**

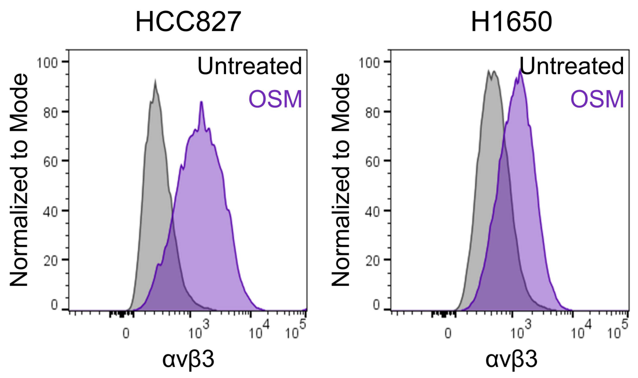

Figure S4. related to Figure 6

A. Pancreatic cancer cell lines (β3-inducible, green; non-inducible, blue) were stimulated with a panel of IL6 family cytokines and TGFβ1 for 72 hours. Cell lysates were immunoblotted to detect expression of integrin β3 and STAT3 pY705.

B. Plots show publicly available ATAC-seq data for a panel of 3 β3-inducible cell lines (green) and 3 non-inducible cell lines (blue), demonstrating differences in chromatin accessibility at the two STAT3 enhancer sites identified for the *ITGB3* gene, GH17J047239 and GH17J047305.

C. Graphs show flow cytometry analysis for integrin αvβ3 cell surface expression for HCC827 and H1650 human lung cancer cell lines that were treated with vehicle control (black) or OSM (purple) for 96 hours.

Figure S5. related to Figure 6

A **HDAC inhibitor enables non-inducible cells to gain β3 in response to Hypoxia**

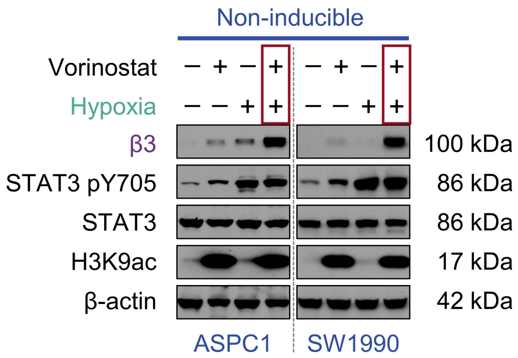

B **H3K27ac ChIP-PCR: H3K27 acetylation occurs at enhancers for *ITGB3* in non-inducible cells following Vorinostat treatment**

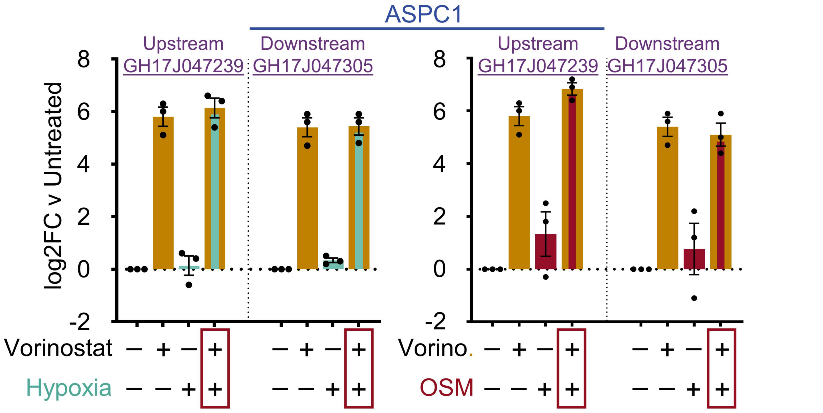

C **Differential expression analysis on 5 cell lines (inducible) vs. 5 cell lines (non-inducible)**

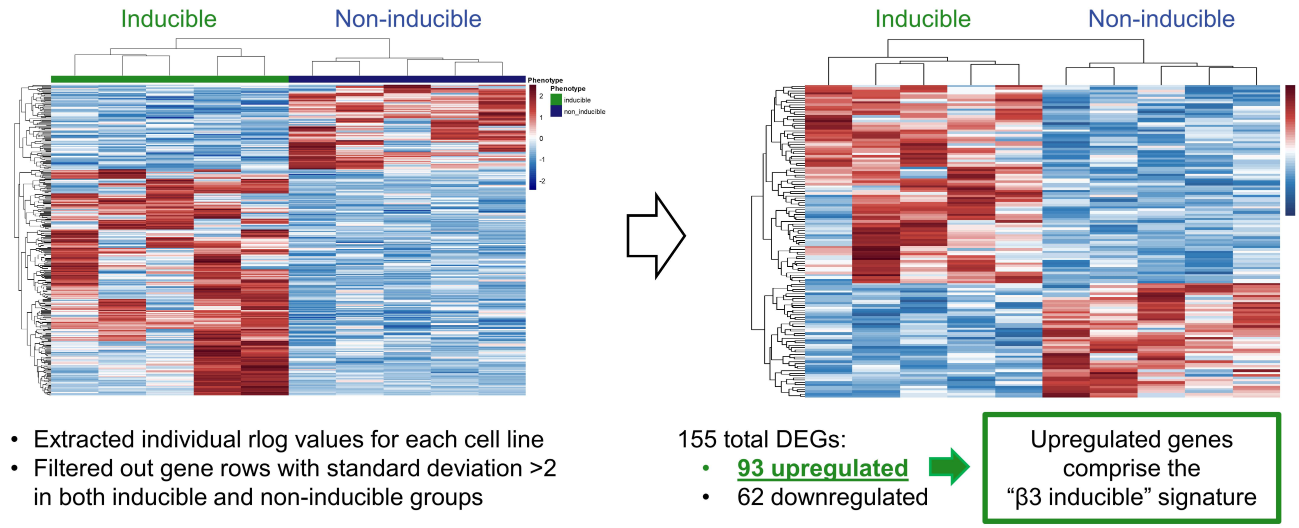

D **Inducible and non-inducible lines show distinct patterns of differential expression**

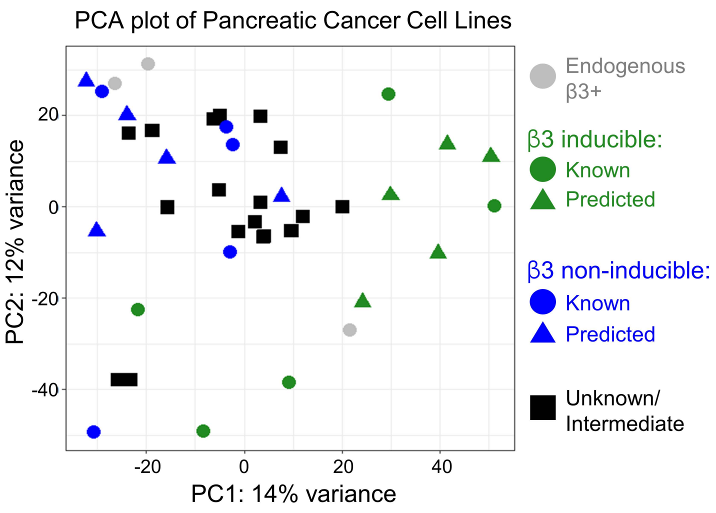

E **β3 inducible signature predicts metastatic potential of CCLE pancreatic cancer cell lines**

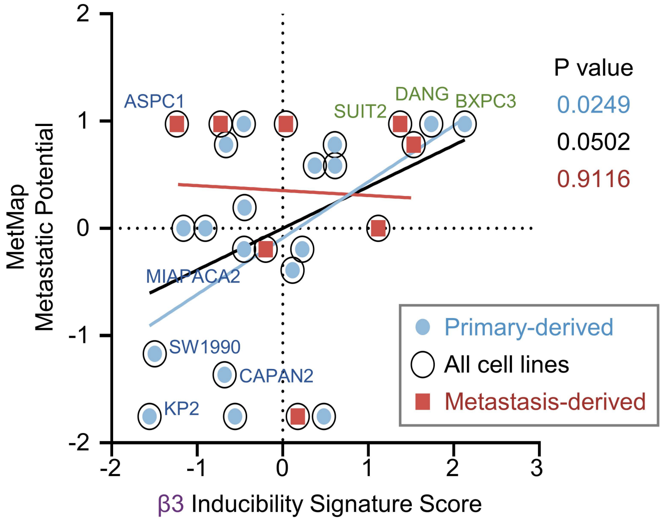

Figure S5. related to Figure 6

A. Two non-inducible cell lines (ASPC1 and SW1990) were untreated or treated for 72 hours with hypoxia (1% oxygen) and the HDAC inhibitor Vorinostat (5μM) alone or in combination, then lysates were blotted to detect β3, STAT3 pY705, total STAT3, H3K9ac, and β-actin. Blots are representative of n = 3 independent experiments per cell line per condition.

B. A non-inducible cell (ASPC1) was untreated or treated with hypoxia (1% oxygen) and the HDAC inhibitor Vorinostat (5μM) alone or in combination (left). Alternatively, cells were untreated or treated with OSM (10 ng/mL) and the HDAC inhibitor Vorinostat (5μM) alone or in combination (right). H3K27ac at upstream and downstream enhancers for *ITGB3* was measured by ChIP-PCR. Graph depicts mean±SEM log2 fold change relative to untreated control for n = 3 independent experiments.

C. Schematic explains steps used to identify β3 inducible signature.

D. Principal component analysis (PCA) performed on PDAC cell lines available through CCLE. Inducible cell lines are shown in green. Non-inducible cell lines are shown in blue. Cell lines with empirically determined phenotypes are indicated with circles, and cells lines with phenotypes that are predicted using the β3 inducible signature scores are represented with triangles. Cell lines with endogenous expression of β3 or cell lines with intermediate signature scores not assigned a predicted phenotype are shown in gray and black respectively.

E. Pearson correlation analysis was performed between cell lines' MetMap scores and their β3 inducibility scores. Cell lines resected and developed from metastatic sites are represented with red squares and cell lines originating form primary sites are indicated by blue circles.

Figure S6. related to Figure 7

A Individual genomic regions (peaks) identified in both ChIP-seq and the ATAC-seq datasets

GeneHancer ID	Annotation	Protein Coding Genes
GH01J109196	Enhancer	*CELSR2, CFAP276, STRIP1, PRPF38B, SARS1,ELAPOR1*
GH01J084851	Enhancer	*C1orf52, GNG5, LPAR3, BCL10, SSX2IP, MCOLN2*
GH11J085717	Enhancer	*CREBZF, CCDC83, TMEM126A, SYTL2, CCDC89*
GH12J047093	Enhancer	*AMIGO2, PCED1B, RPAP3*
GH14J051543	Enhancer	*GNG2, ABHD12B, FRMD6*
GH17J047239	Enhancer	*EFCAB13, ITGB3, MYL4, RPS7P11, TBKBP1*
GH03J134008	Enhancer	*SLCO2A1, RAB6B, C3orf36*
GH03J100525	Enhancer	*ADGRG7, TMEM45A*
GH05J169694	Enhancer	*DOCK2, INSYN2B, SPDL1*
GH05J035882	Enhancer	*IL7R, CAPSL, LMBRD2*
GH07J092746	Enhancer	*CDK6, FAM133B, KRIT1, PEX1, VPS50*
GH08J133176	Enhancer	*CCN4, TG, PHF20L1, SLA, TMEM71, ST3GAL1*
GH09J134601	Enhancer	*BRD3, WDR5, COL5A1, RXRA*
GH09J134625	Enhancer	*RXRA, COL5A1*

B Initial correlation analysis of genes associated with cross-referenced peaks identified in Fig 7C

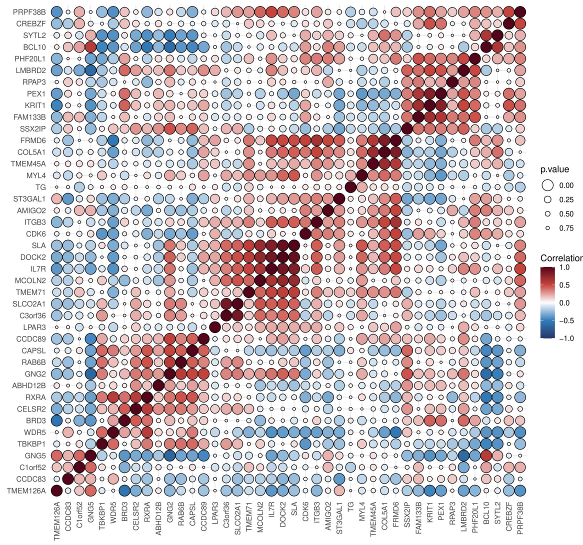

C Validation of *STRESS* signature in Inducible and Non-inducible cells

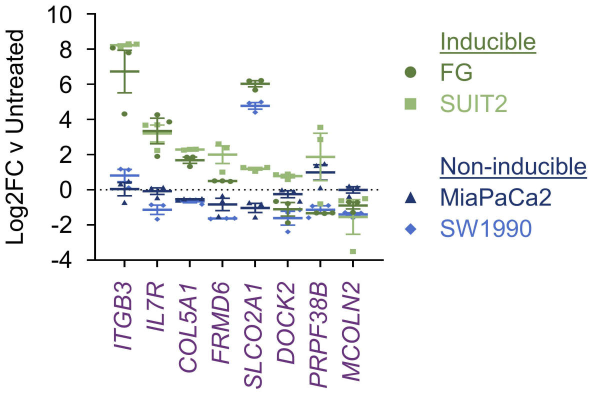

D *STRESS* signature does not correlate with PDAC subtypes

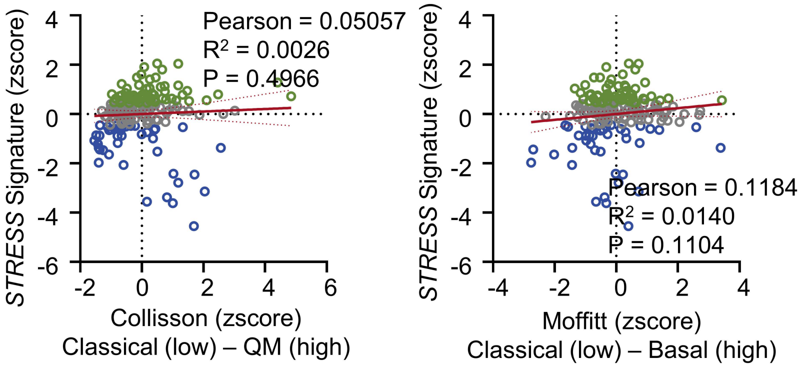

E *STRESS* signature does not overlap with previously reported PDAC subtype groups

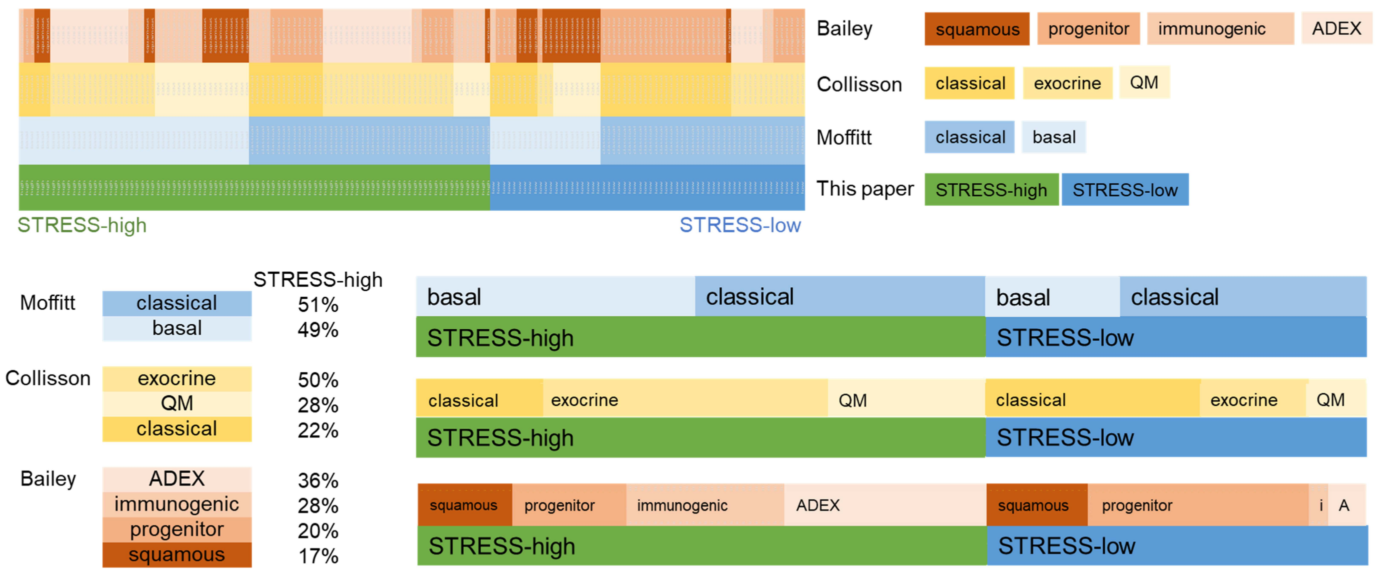

Figure S6. related to Figure 7

A. List of GeneHancer IDs that showed STAT3 pY705 enrichment in ChIP-seq screen, along with the corresponding list of protein coding genes for each enhancer.

B. Initial correlation analysis of genes associated with cross-referenced peaks identified in Figure 7C in The Cancer Genome Atlas (TCGA) PAAD pancreatic cancer cohort.

C. Validation of *STRESS* signature genes in two inducible (FG and SUIT2; green) and two non-inducible (MiaPaCa2 and SW1990; blue) cancer cell lines. Cells were untreated or stimulated with OSM (10 ng/mL) for 72 hours then processed for RT-qPCR analysis. Graph depicts mean±SEM log2 fold change relative to untreated control for n = 3 independent experiments per cell line.

D. Pearson correlation comparing expression of the *STRESS* signature genes to the Collisson and Moffitt signatures in The Cancer Genome Atlas (TCGA) PAAD pancreatic cancer cohort.

E. Color map depicting the relationship between the *STRESS* signature and previously reported PDAC subtype groups in The Cancer Genome Atlas (TCGA) PAAD pancreatic cancer cohort.

Figure S7. related to Figure 7

A **No overlapping genes between the *STRESS* signature and previously reported molecular subtypes**

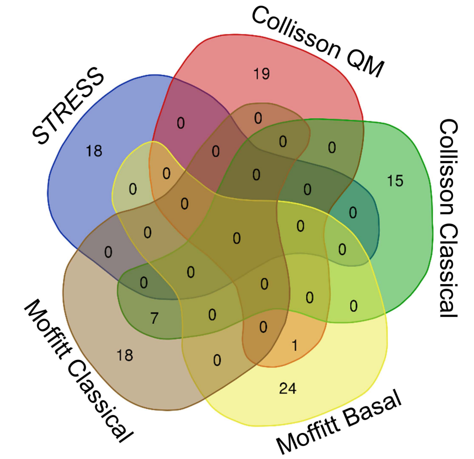

B ***STRESS* signature correlates with β3 inducibility signature**

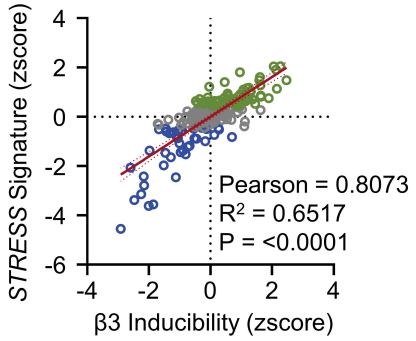

C **No overlapping genes between the *STRESS* and β3 inducible signatures**

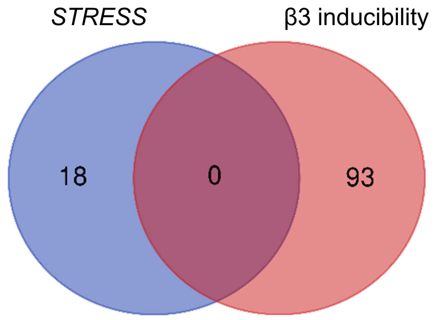

D ***STRESS* and molecular subtype signatures are less predictive for overall survival (OS)**

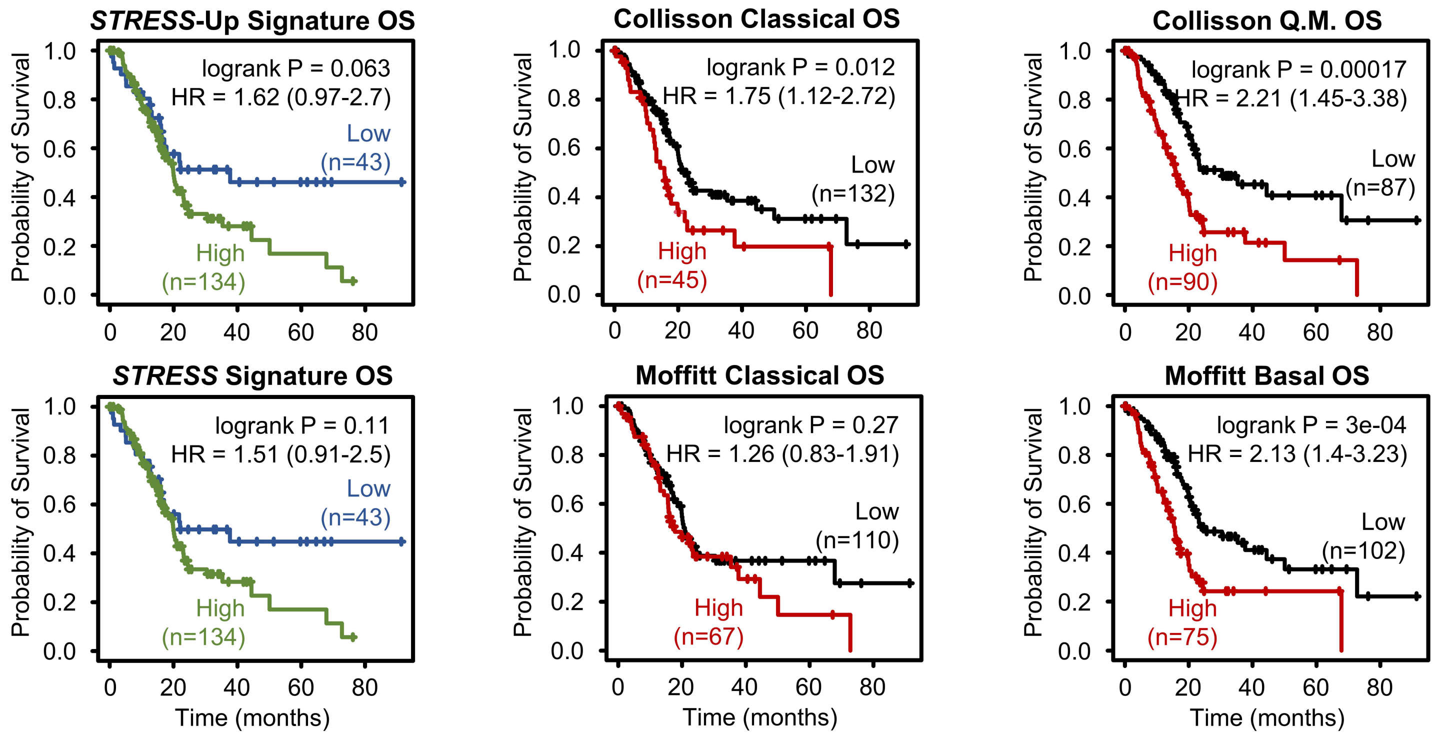

E ***STRESS* signaling is high in TCGA PAAD and LUAD Patient Cohorts**

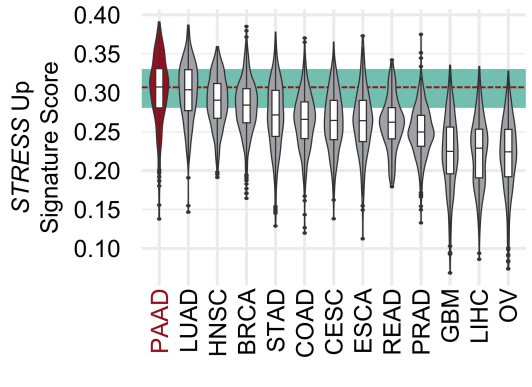

F **Relapse-free survival analyses to compare the *STRESS* and molecular subtype gene signatures between PDAC and other cancer types**

	Hazard Ratios				*P-Values*			
TCGA datasets:	PDAC	LUAD	Rectal	Breast	PDAC	LUAD	Rectal	Breast
***STRESS* Signature (up genes)**	16.90	1.40	2.80	1.60	**0.00**	0.20	0.19	0.05
***STRESS* Signature (all genes)**	6.10	1.40	2.80	1.40	**0.00**	0.17	0.19	0.11
Moffitt Classical	4.30	1.50	0.00	0.40	0.12	0.73	0.13	**0.00**
Collisson Classical	4.20	1.70	0.00	0.70	**0.01**	**0.01**	0.09	0.06
Collisson Quasi-Mesenchymal	3.70	1.50	0.30	1.60	**0.00**	0.06	0.27	**0.03**
Moffitt Basal	3.40	1.70	3.70	0.50	**0.00**	**0.01**	**0.01**	**0.01**

Figure S7. related to Figure 7

A. Venn overlap analysis shows overlap of one gene (*S100A2*) between Collisson quasi-mesenchymal (QM) and Moffitt basal signatures, and seven genes (*TFF3, TSPAN8, LGALS4, CEACAM6, AGR2, ST6GALNAC1, TFF1*) common to Collisson classical and Moffitt classical. None of the 18 genes comprising the *STRESS* signature overlap with these benchmark molecular subtypes.

B. Pearson correlation comparing expression of the *STRESS* signature genes to the β3-inducible signature genes in The Cancer Genome Atlas (TCGA) PAAD pancreatic cancer cohort.

C. Venn overlap analysis shows no overlap of genes in the *STRESS* vs. β3-inducible signatures.

D. KM Plotter analysis of overall survival (OS) for pancreatic cancer patients according to the *STRESS* or *STRESS*-Up signatures compared with previously reported molecular subtypes of pancreatic cancer (Collisson and Moffitt signatures).

E. Violin plots show the distribution of patient *STRESS*-UP Signaling gene set scores for RNA sequencing of patient tumors in The Cancer Genome Atlas (TCGA).

F. List of hazard ratios (HR) and corresponding P-values for the effect of the STRESS, Collisson, or Moffitt signatures on relapse-free survival (RFS) for multiple TCGA datasets, including pancreatic cancer (PDAC), lung adenocarcinoma (LUAD), rectal cancer, and breast cancer.

Table S1. related to Figure 1

45/106 STAT3 “stress response genes” from ChIP-seq analyses linked to poor survival for PDAC

Known STAT3 Target	Gene Name	GeneHancer ID	Genomic Region	RFS p.value	RFS HR	Hypoxia padj rank	OSM padj rank	Overall rank	Hypoxia Log2FC	Hypoxia padj	OSM log2FC	OSM padj
No	*ITGB3*	GH17J047239	Enhancer	0.0002	10.59	13	1	6	0.86	8.4E-07	2.38	3.0E-30
No	*ZBTB38*	GH03J141322	Promoter	0.0002	5.27	4	13	7	2.47	9.1E-10	3.31	8.8E-10
No	*KCNJ15*	GH21J038266	Promoter	0.0051	3.23	32	27	19	1.59	2.6E-04	2.39	1.7E-06
No	*STN1*	GH10J103892	Enhancer	0.0065	3.29	26	62	40	1.79	5.9E-05	1.63	1.2E-03
No	*RP1*	GH08J054628	Enhancer	0.0010	5.19	87	16	49	1.37	2.3E-02	2.62	1.1E-08
No	*TBPL2*	GH14J055446	Enhancer	0.0280	2.49	55	64	62	0.60	3.1E-03	2.05	1.4E-03
No	*PHLDB2*	GH03J111901	Enhancer	0.0458	3.98	29	96	64	1.26	9.4E-05	0.98	2.8E-02
No	*FAR2*	GH12J029239	Enhancer	0.0268	2.82	67	81	78	1.08	7.3E-03	0.86	1.0E-02
No	*CD44*	GH11J035157	Promoter	0.0002	4.52	74	89	88	1.34	1.3E-02	1.87	2.3E-02
No	*STN1*	GH10J103878	Enhancer	0.0065	3.29	78	91	93	1.50	1.5E-02	1.58	2.5E-02
Yes	*NOX4*	GH11J089379	Enhancer	0.0283	2.75	2	7	2	1.47	1.9E-20	1.49	7.5E-13
Yes	*TPBG*	GH06J082407	Enhancer	0.0194	4.82	10	40	14	1.76	5.4E-07	2.45	1.8E-05
Yes	*TP63*	GH03J189571	Enhancer	0.0396	3.43	35	21	16	1.52	3.7E-04	2.42	2.5E-07
Yes	*CEP128*	GH14J080621	Enhancer	0.0064	6.24	50	9	19	0.72	1.5E-03	2.22	6.7E-12
Yes	*BMPR1B*	GH04J095066	Enhancer	0.0123	3.54	15	47	21	1.19	3.4E-06	1.83	1.9E-04
Yes	*SMAD3*	GH15J067113	Promoter	0.0008	14.15	46	18	23	1.35	9.8E-04	2.34	3.1E-08
Yes	*MMP13*	GH11J102950	Enhancer	0.0233	2.58	9	55	23	1.11	1.3E-07	1.27	5.1E-04
Yes	*SLC9B2*	GH04J103068	Enhancer	0.0073	3.14	56	12	26	1.71	3.6E-03	2.57	3.1E-10
Yes	*IGF1R*	GH15J098887	Promoter	0.0045	3.13	20	48	26	0.90	1.8E-05	1.12	2.5E-04
Yes	*ITGB6*	GH02J160090	Enhancer	0.0001	4.33E+08	23	56	34	1.74	3.7E-05	1.98	5.2E-04
Yes	*SEMA3A*	GH07J084088	Enhancer	0.0003	9.63	31	50	35	1.31	2.2E-04	2.09	2.7E-04
Yes	*EXT1*	GH08J118141	Enhancer	0.0030	3.16E+08	44	42	39	0.76	8.8E-04	1.31	4.1E-05
Yes	*TGFBI*	GH05J136013	Enhancer	0.0039	3.21	11	79	41	1.64	6.5E-07	1.40	8.4E-03
Yes	*IL7R*	GH05J035882	Enhancer	0.0247	4.05	72	22	43	0.81	1.1E-02	2.03	3.2E-07
Yes	*DNMBP*	GH10J099962	Enhancer	0.0240	3.78	36	58	43	1.31	3.9E-04	1.50	5.3E-04
Yes	*FHL2*	GH02J105402	Enhancer	0.0381	2.76	70	26	45	0.53	9.9E-03	1.02	5.6E-07
Yes	*SERPINB4*	GH18J063641	Enhancer	0.0321	3.51	83	14	46	0.70	2.0E-02	2.24	1.9E-09
Yes	*GALNT2*	GH01J230111	Enhancer	0.0084	3.91	73	29	48	0.64	1.2E-02	1.37	1.8E-06
Yes	*PIK3R4*	GH03J130773	Enhancer	0.0069	4.98	85	19	51	0.46	2.3E-02	1.68	3.9E-08
Yes	*PACS1*	GH11J066111	Promoter	0.0497	2.55	43	77	63	1.40	8.1E-04	1.43	5.2E-03
Yes	*IL1RAP*	GH03J190562	Enhancer	0.0008	8.27	54	71	64	1.59	2.3E-03	1.19	3.5E-03
Yes	*YAP1*	GH11J102222	Enhancer	0.0001	5.06	28	97	64	0.66	8.9E-05	0.52	2.9E-02
Yes	*CDC42EP3*	GH02J037633	Promoter	0.0242	2.6	92	34	67	1.16	2.7E-02	3.16	4.9E-06
Yes	*EGFR*	GH07J054994	Enhancer	0.0010	13.87	42	85	69	2.52	7.7E-04	1.35	1.7E-02
Yes	*USP43*	GH17J009676	Enhancer	0.0167	3.16	96	32	70	0.99	3.3E-02	2.07	3.2E-06
Yes	*SMC6*	GH02J017615	Enhancer	0.0015	14.82	71	63	72	0.96	1.1E-02	1.59	1.3E-03
Yes	*SORD*	GH15J045068	Enhancer	0.0477	2.84	81	53	72	0.76	1.8E-02	1.57	4.1E-04
Yes	*PMCH*	GH12J102198	Promoter	0.0010	3.89	65	73	75	1.55	7.1E-03	2.44	3.9E-03
Yes	*SLC37A3*	GH07J140314	Enhancer	0.0144	3.58	49	103	81	1.56	1.3E-03	0.88	4.2E-02
Yes	*PANX1*	GH11J094127	Promoter	0.0004	4.12	62	95	85	1.75	6.3E-03	0.81	2.7E-02
Yes	*REPS2*	GH0XJ017077	Enhancer	0.0011	8.49	93	76	93	1.56	2.9E-02	1.76	4.4E-03
Yes	*MECOM*	GH03J169309	Enhancer	0.0004	4.23	89	80	93	0.96	2.5E-02	1.66	9.9E-03
Yes	*MCC*	GH05J113161	Enhancer	0.0303	3.72	88	88	98	0.87	2.5E-02	0.83	2.3E-02
Yes	*SDR16C5*	GH08J056322	Enhancer	0.0023	12.11	84	94	101	0.85	2.1E-02	0.89	2.6E-02
Yes	*MET*	GH07J116677	Enhancer	0.0001	6.11	105	86	104	1.13	4.6E-02	1.92	1.7E-02

Table S2. related to Figure 4

Clinical spectrum of disease in i-KPC and i-KPC+β3KO mice

Experiment	Mouse	Sex	days to moribund	%Diff (Well / Mod. / Poor)	Pathology
Survival/Late-stage	i-KPC #192	F	64	-	-
Survival/Late-stage	i-KPC #181	M	67	0 / 100 / 0	-
Survival/Late-stage	i-KPC #182	F	69	-	-
Survival/Late-stage	i-KPC #203	F	84	0 / 0 / 100	-
Survival/Late-stage	i-KPC #260	M	94	-	-
Survival/Late-stage	i-KPC #186	M	100	0 / 0 / 100	-
Survival/Late-stage	i-KPC #178	F	105	0 / 4.6 / 95.4	-
Survival/Late-stage	i-KPC #187	M	106	0 / 0 / 100	-
Survival/Late-stage	i-KPC #190	M	110	13.1 / 0 / 86.9	-
Survival/Late-stage	i-KPC #246	F	112	0 / 100 / 0	-
Survival/Late-stage	i-KPC #256	F	112	0 / 0 / 100	-
Survival/Late-stage	i-KPC #177	F	143	-	-
Survival/Late-stage	i-KPC #180	M	193	16.8 / 0 / 83.2	-
Survival/Late-stage	i-KPC #188	M	193	0 / 0 / 100	-
Survival/Late-stage	i-KPC+β3KO #148	M	101	100 / 0 / 0	-
Survival/Late-stage	i-KPC+β3KO #90	F	108	86.6 / 13.4 / 0	-
Survival/Late-stage	i-KPC+β3KO #102	M	117	16.6 / 8.2 / 75.1	-
Survival/Late-stage	i-KPC+β3KO #101	F	123	91.6 / 8.4 / 0	-
Survival/Late-stage	i-KPC+β3KO #140	F	132	-	-
Survival/Late-stage	i-KPC+β3KO #103	M	146	0 / 9.3 / 90.7	-
Survival/Late-stage	i-KPC+β3KO #157	F	147	37.8 / 62.2 / 0	-
Survival/Late-stage	i-KPC+β3KO #151	M	149	50.7 / 49.3 / 0	-
Survival/Late-stage	i-KPC+β3KO #149	M	155	80.4 / 19.6 / 0	-
Survival/Late-stage	i-KPC+β3KO #93	F	175	0 / 0 / 100	-
Survival/Late-stage	i-KPC+β3KO #147	M	176	0.3 / 0 / 99.7	-
Survival/Late-stage	i-KPC+β3KO #99	F	200	0 / 0 / 100	-
Survival/Late-stage	i-KPC+β3KO #95	M	208	73.6 / 26.4 / 0	-
Survival/Late-stage	i-KPC+β3KO #100	F	211	100 / 0 / 0	-
Early Stage (60 days)	i-KPC #425	F	-	-	W, 1.B3
Early Stage (60 days)	i-KPC #426	F	-	-	WMP, 3.B3
Early Stage (60 days)	i-KPC #428	F	-	-	WM, 2.B3
Early Stage (60 days)	i-KPC #436	M	-	-	P, 3.B3
Early Stage (60 days)	i-KPC #437	M	-	-	WMP, 3.B3
Early Stage (60 days)	i-KPC+β3KO #401	F	-	-	H, 0.B3
Early Stage (60 days)	i-KPC+β3KO #402	F	-	-	pan, 0.B3
Early Stage (60 days)	i-KPC+β3KO #125	F	-	-	H, 0.B3
Early Stage (60 days)	i-KPC+β3KO #127	F	-	-	H, 0.B3
Early Stage (60 days)	i-KPC+β3KO #128	M	-	-	H, 0.B3
Early Stage (60 days)	i-KPC+β3KO #406	M	-	-	H, 0.B3

Pathology: Well (W), Moderate (M), Poor (P), no integrin β3 expression (0.β3) to high β3 expression (3.β3).

Table S3. related to Figure 7

Gene sets of common PDAC subtype signatures and *STRESS* and β3 inducible signatures

Names	total	elements
Collisson QM<br>Moffitt Basal	1	*S100A2*
Collisson Classical<br>Moffitt Classical	7	*TFF3 TSPAN8 LGALS4 CEACAM6 AGR2 ST6GALNAC1 TFF1*
STRESS	18	*SLCO2A1 IL7R COL5A1 RXRA CAPSL RAB6B MCOLN2 PEX1 FAM133B TMEM71 SSX2IP SLA FRMD6 ITGB3 DOCK2 PRPF38B LMBRD2 KRIT1*
Collisson QM	19	*PMAIP1 TWIST1 PAPPA SLC2A3 SLC16A1 KRT14 FAM26F HK2 GPM6B LOX AHNAK2 AIM2 CKS2 SLC5A3 NT5E FERMT1 CAV1 HMMR PHLDA1*
Collisson Classical	15	*CAPN8 CEACAM5 ATP10B FXYD3 GPX2 SDR16C5 S100P TMEM45B ELF3 TOX3 GPRC5A FOXQ1 PLS1 MUC13 ERBB3*
Moffitt Basal	24	*ANXA8L2 CTSL2 GPR87 UCA1 LY6D KRT6A LEMD1 CST6 SERPINB3 AREG SLC2A1 KRT15 SCEL SPRR1B KRT17 DHRS9 VGLL1 TNS4 KRT7 FGFBP1 SERPINB4 KRT6C FAM83A SPRR3*
Moffitt Classical	18	*BTNL8 FAM3D LOC400573 TFF2 MYO1A ANXA10 PLA2G10 KRT20 CTSE REG4 VSIG2 CYP3A7 CLRN3 AGR3 ATAD4 SPINK4 LYZ CDH17*
β3 inducible sig.	93	*RPLP0P2 CD24P4 ITGA1 SV2C CLPB FOXC2 AKR1B10 HSD11B2 LINC01234 SEMA6D HOXD3 PAK6 AC138305 PAX5 HMGB3P32 RHOV PDGFRA TMEM255A NLRP7 DTNA CLCA3P CYP19A1 ZNF385A GCN1 LINC01399 ZNF697 RASD1 PITX2 GPRIN3 LRIG1 EHHADH NCF1C NCF1B ENAM AC128689 TRAV13-2 SAMD5 P2RY1 CD69 AC073365 PDGFRL CCBE1 AHSG AC104083 LCK CD6 KCNMB4 SOX2 NCF1 ADRA2A ALPL SORD2P CLPSL1 KIF12 LINC01215 MCEMP1 ADGRV1 NAMPTP1 SNX31 RASGRF2 NAGS CYP2C9 BMPR1B CFHR3 CXCL9 SHC4 AL355076 AC040174 LINC00668 ASB2 CD79A BVES AC084783 TNFRSF8 PI15 BACH2 NUGGC UST TMC8 ADGRG7 SALL4 AC090204 CPOX SPINK5 AQP3 HEY2 RASSF9 AR DMC1 BCL11A IKZF3 XKR9 BTK*
